# Observational prospective study of social media, smartphone use and self-harm in a clinical sample of young people: study protocol

**DOI:** 10.1136/bmjopen-2022-069748

**Published:** 2023-02-01

**Authors:** Amanda Bye, Ben Carter, Daniel Leightley, Kylee Trevillion, Maria Liakata, Stella Branthonne-Foster, Grace Williamson, Zohra Zenasni, Rina Dutta

**Affiliations:** 1 Psychological Medicine, King’s College London Institute of Psychiatry Psychology and Neuroscience, London, UK; 2 Biostatistics and Health Informatics, King’s College London Institute of Psychiatry Psychology and Neuroscience, London, UK; 3 King’s Centre for Military Health Research, King’s College London Institute of Psychiatry Psychology and Neuroscience, London, UK; 4 Health Service and Population Research, King’s College London Institute of Psychiatry Psychology and Neuroscience, London, UK; 5 School of Electronic Engineering and Computer Science, Queen Mary University of London, London, UK; 6 The Alan Turing Institute, London, UK; 7 No affiliations, London, UK; 8 South London and Maudsley NHS Foundation Trust, London, UK

**Keywords:** Suicide & self-harm, Child & adolescent psychiatry, MENTAL HEALTH, STATISTICS & RESEARCH METHODS, QUALITATIVE RESEARCH

## Abstract

**Introduction:**

Young people are the most frequent users of social media and smartphones and there has been an increasing speculation about the potential negative impacts of their use on mental health. This has coincided with a sharp increase in the levels of self-harm in young people. To date, studies researching this potential association are predominantly cross-sectional and reliant on self-report data, which precludes the ability to objectively analyse behaviour over time. This study is one of the first attempts to explore temporal patterns of real-world usage prior to self-harm, to identify whether there are usage patterns associated with an increased risk.

**Methods and analysis:**

To study the mechanisms by which social media and smartphone use underpin self-harm in a clinical sample of young people, the Social media, Smartphone use and Self-harm in Young People (3S-YP) study uses a prospective, observational study design. Up to 600 young people aged 13–25 years old from secondary mental health services will be recruited and followed for up to 6 months. Primary analysis will compare real-world data in the 7 days leading up to a participant or clinician recorded self-harm episode, to categorise patterns of problematic usage. Secondary analyses will explore potential mediating effects of anxiety, depression, sleep disturbance, loneliness and bullying.

**Ethics and dissemination:**

This study was approved by the National Research Ethics Service, London - Riverside, as well as by the Joint Research and Development Office of the Institute of Psychiatry, Psychology and Neuroscience and South London and Maudsley NHS Foundation Trust (SLaM), and the SLaM Clinical Research Interactive Search (CRIS) Oversight Committee. The findings from this study will be disseminated through peer-reviewed scientific journals, conferences, websites, social media and stakeholder engagement activities.

**Trial registration number:**

NCT04601220.

**Strengths and limitations of this study**

- The Social media, Smartphone use and Self-harm in Young People (3S-YP) study uses a novel prospective, observational strategy to investigate the patterns of real-world social media and smartphone usage prior to episodes of self-harm in a clinical youth sample.
- Co-production with youth experts by experience, including coauthor (SB-F), a leading national UK youth mental health charity—YoungMinds, and a steering group comprising of experts in the field of self-harm and suicide prevention will ensure acceptability and relevance, as well as facilitate dissemination and impact.
- Due to the online nature of the study, participants need to be able to independently engage with the digital tools and study documentation that is only available in the English language.
- The flexible approach to participation regarding which data young people choose to share, will ensure our methods are acceptable and promote autonomy among participants, though it will increase the risk of missing and inconsistent data which could compromise the validity of the results and interpretations made.
- Mixed model analyses, state-of-the-art natural language processing methods combined with thematic analysis, will be used to analyse social media metadata, textual and imagery data and smartphone metadata.

## Introduction

Self-harm is characterised as any behaviour where an individual causes damage or injury to their body in response to distress. Traditionally, research has focused more on severe cases of self-harm, namely cutting or self-poisoning, rather than a broader spectrum of behaviour, including burning, scratching or hitting oneself. Self-harm in young people is a significant public health concern, with findings indicating that more than 15% of the young people report engaging in self-harm in the community, coupled with high rates of repetition and elevated risk of suicide.^1–3^ Prevalence figures are likely to underestimate the true scale of the problem, given young people are often reluctant to disclose and seek professional help owing to fear of stigma.^4^ Furthermore, epidemiological studies tend to rely on hospital presentations and only reflect those cases of self-harm that are brought to medical attention.^5^ Whereas community and school-based studies collect self-reported retrospective data. Therefore, while capturing a wider range of self-harm behaviours, they lack information about the specific timing and nature of non-help seeking self-harm.

Reasons for self-harm are complex and multifaceted, however there has been an increasing speculation about the potential negative impacts of social media and smartphone use on youth mental health.^6^ Young people are the most frequent users of social media and smartphones, with 91% of 12–15 year olds using social media and 98% of 13-year olds owning a smartphone in the UK.^7^ Over the last decade, social media and smartphones have altered how young people spend their time and interact socially with peers.^8^ This coincides with a sharp increase in the levels of self-harm, particularly among adolescent women.^9^ Given the widespread use of social media and smartphones, understanding the implications for youth mental health is of paramount importance.

Social media platforms are often used by people with mental health problems to share personal experiences and seek support.^10^ Feedback received can be perceived as supportive by the individual.^11^ Thus, social media use can alleviate loneliness and social isolation that is common among young people with mental health problems as it can provide opportunities for strengthening existing friendships and developing new social connections, however, it can also exacerbate loneliness if excessive time is spent online rather than time that would be otherwise spent interacting socially offline.^12 13^

Online forums have been a cause for particular concern as users may be exposed to harmful self-harm content and online discussions that normalise behaviours and discourage help-seeking, which may be associated with increased suicidal ideation found among forum users compared with other forms of social media user.^10^ Other studies have found significant associations between cyberbullying victimisation and self-harm in young people, and to a lesser extent, cyberbullying perpetration, likely perpetuated by the anonymous nature of social media.^14^ Young people engaging in self-harm behaviours may also be more likely to engage in risky online behaviour compared with their peers.^15^ More recent findings indicate that while for some young people and in certain circumstances, social media can have a positive or neutral effect, for others it can be harmful and so more research is needed to explore the effects of different types of social media use on an individual level.^16 17^

Research on the effects on mental health of excessive screen time in general is more conflicting, possibly relating to inconsistencies in measurements and definitions employed.^18–20^ Sohn *et al*,^20^ found evidence of an association between problematic smartphone usage and increased anxiety and depression. Emerging evidence indicates that problematic smartphone usage may be positively linked with suicide ideation and the association may be mediated by emotional intelligence.^21 22^ Furthermore, there is an established literature linking excessive smartphone use and sleep disturbance^23^ and poor sleep has been linked to depression and suicidal ideation in young people,^24^ as well as there being an association between sleep problems and suicidal thoughts and behaviours independent of depression.^25^

Much of the current evidence on the association between social media and smartphone use and mental health and self-harm among young people is limited to associations from cross-sectional studies (eg, Mancinelli *et al*).^26^ Where longitudinal studies are reported, they are based on small samples, are reliant on self-report data and have prolonged periods of time between waves of data collection (eg, Arendt *et al*.)^27^ These approaches have precluded the ability to explore potential mechanisms underlying self-harm behaviour over time using real-world data, in a prospective way. This study is one of the first to attempt to explore the temporal patterns of social media and smartphone use prior to episodes of self-reported and clinician-reported self-harm events, to identify whether there are usage patterns of social media and smartphone use that are associated with increased risk of self-harm. The findings from this study have the potential for generating a wide-ranging impact as well as filling the gaps in current knowledge. The overarching aim of the Social media, Smartphone use and Self-harm in Young People (3S-YP) study is to investigate the mechanisms by which social media and smartphone use underpin self-harm in a clinical sample of young people. Primary objectives are:

- To identify characteristic patterns of social media and smartphone use in the 7 days preceding an episode of self-harm.
- To determine whether characteristic patterns of social media and smartphone use associated with an episode of self-harm, differ from young people who have not had an episode of self-harm.

Secondary objectives are to investigate whether the association is mediated by sleep disturbance, depression, anxiety, loneliness and bullying.

## Methods and analysis

### Study design

This study is a prospective 6-month cohort study and has been reported according to the Strengthening the Reporting of Observational Studies in Epidemiology checklist for cohort studies (see online supplemental additional file 1) (protocol V.1.4, 21/07/2022).

10.1136/bmjopen-2022-069748.supp1 Supplementary data

### Study setting

Young people (aged 13–25 years) will be recruited from South London and Maudsley NHS Foundation Trust (SLaM). SLaM is one of the largest mental healthcare providers in Europe, providing secondary mental healthcare to a local population of approximately 1.3 million residents as well as national and specialist services.

### Participant selection

Young people will be identified via one of the following selection methods:

1. SLaM’s Consent for Contact (C4C) patient research participation register of patients who have given prior consent to researchers with ethically approved studies to access their health records and contact them for the purposes of recruitment to research for which they are eligible.^28^ A researcher who is independent of the study team and who works for the National Institute for Health Research Biomedical Research Centre within SLaM, will apply the eligibility criteria to the register using the Clinical Research Interactive Search (CRIS) system to screen for potentially eligible young people. The CRIS system is a case register that contains de-identified information extracted from the Trust’s electronic health record (EHR) system.^29^ The study team will then be supplied with the Trust-identifying details of potentially eligible young people so they can check their EHRs in detail for clinical eligibility. For eligible young people, the study team will inform the care coordinator of the intention to approach prior to initiating any contact. This is so that care coordinators can advise if it is not appropriate for a young person under their care to be approached for the study.
2. If the above yields insufficient numbers, we will invite clinicians to refer young people under their care directly to the study team. We will attend team meetings to explain the study and eligibility criteria to clinicians. Clinicians will screen young people under their care for eligibility and will contact eligible young people (and a parent or carer with parental responsibility for young people aged 13–15 years old) to explain the study and seek permission to share their contact details with the study team.

### Eligibility criteria

Inclusion and exclusion criteria are reported in table 1. No recruitment quotas will be imposed, with respect to any participant characteristics. To reduce the risk of participant selection bias, all eligible young people will be invited to participate until the recruitment target is met.

**Table 1:** Eligibility criteria

Inclusion criteria	Identified via SLaM’s C4C patient research participation register or referral to the study team by their clinician.
	Aged 13–25 years old at the time of study approach.
	Accessed mental health services at SLaM in the last 12 months.
	Has capacity to consent (and an adult with parental responsibility for young people aged 13–15 years old). Mental capacity will be assumed unless evidence from a clinician or during contact with the study team suggests otherwise.
Exclusion criteria	Unable to complete the questionnaires via the study software application or online survey platform.
	Admitted to an inpatient psychiatric ward, sectioned under the Mental Health Act or in prison at the time of approach.
	Clinician advises it is not appropriate to approach.

C4C, Consent for Contact; SLaM, South London and Maudsley NHS Foundation Trust.

### Sample size calculation

In a recent national community survey, the reported annual age-sex incidence of self-harm for young people aged 16–18 years old was 36.4% (95% CI 22.4 to 52.2) for men and 43.5% (95% CI 35.5 to 51.7) for women.^3^ Based on uptake of SLaM C4C patient research participation register, we estimate that if 600 young people consent to take part, 480 will be followed up during a 6-month period, resulting in at least 150 with an episode of self-harm. Using the most conservative estimates, if approximately 22.5% of those who do not display characteristic problematic patterns of social media usage (or smartphone usage) have an episode of self-harm and this increases to 35% in those identified with problematic usage in the previous week, we expect 86% power to detect a 12.5% increase in self-harm at 5% significance, assuming 480 young people are followed up.

### Recruitment method and study procedures

**Figure 1:**
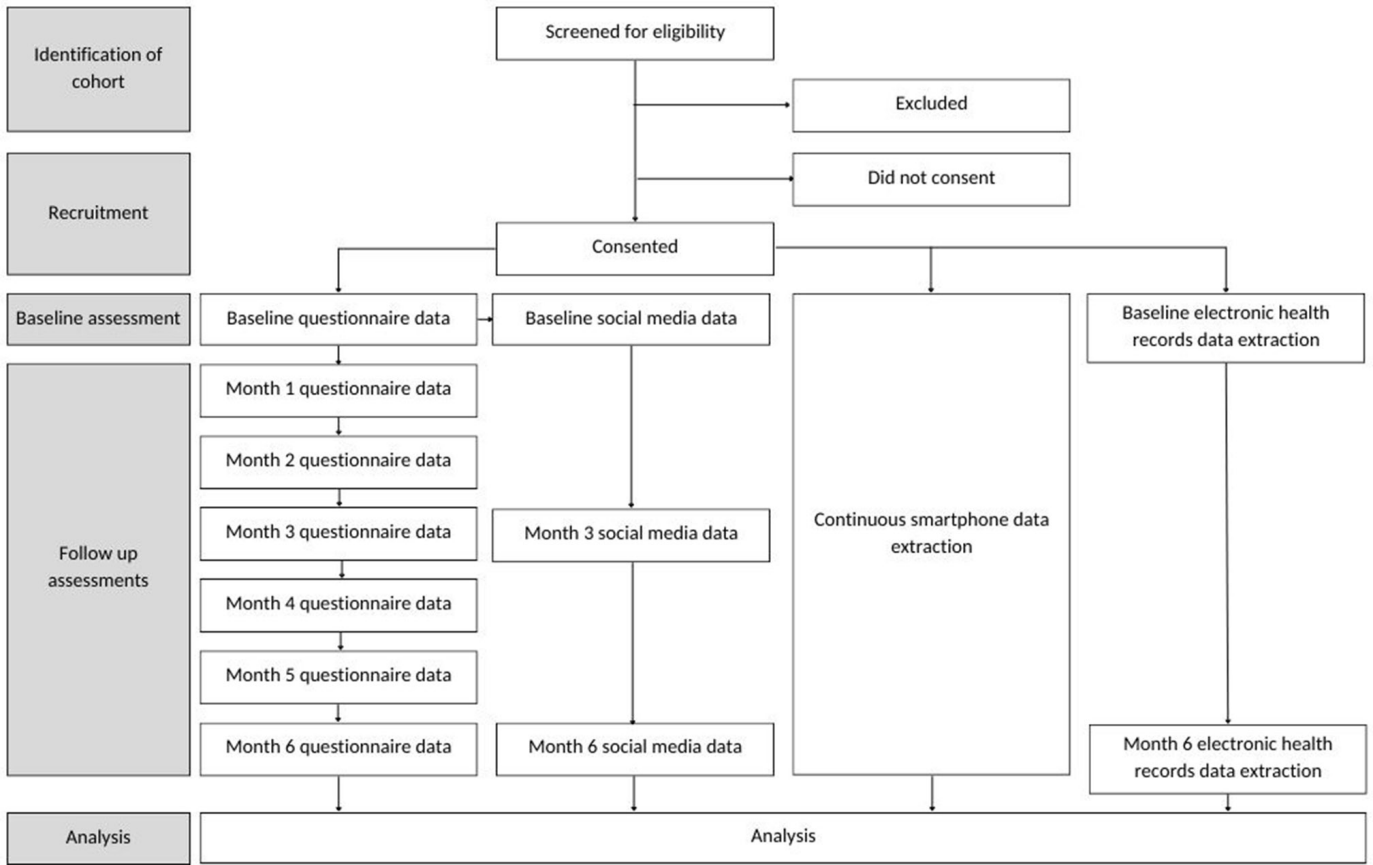
Participant flowchart.

The study team will contact eligible young people (and/or an adult with parental responsibility for young people aged 13–15 years old) by telephone, text or email to invite the young person to take part. They will be sent a unique link to the study website which will host the participant information and online consent and assent forms as well as being offered the option of a face-to-face or virtual meeting if needed. Young people aged 16–25 years old who want to take part, will be asked to confirm their consent via an online form. This will include separate consent for the following types of data collection: questionnaires, EHRs, social media and smartphone data; choosing not to consent to the latter three will not preclude participation. For young people aged 13–15 years old, an adult with parental responsibility will first be asked to confirm their consent via an online form, following which the young person will be asked to confirm their assent. A maximum of three contact attempts will be made to non-responders. The flow of participants through the study will be documented in a flowchart (see figure 1 - Participant flowchart).

Participants will then instal the study software application on their smartphone device and complete the baseline questionnaires. The study software application has been designed to continuously extract smartphone metadata and to deliver the questionnaires for the follow-up period (up to 6 months, reduced from 12 months following an initial pilot phase). Tables 2 and 3 outline data collection measures over the 6-month follow-up period related to primary and secondary objectives and other measurements. Participants who choose not to provide smartphone metadata (or use the study software application for any other reason) will be provided with a unique link to an online survey platform—Qualtrics (https://www.qualtrics.com)—to complete the questionnaires according to the same schedule. Participants will receive a reminder (an automated push notification and/or a prescheduled standardised text message and/or email) on the day a questionnaire is due, followed by a second reminder 7 days later if incomplete. The final set of questionnaires may also be sent via post. Participants who consent to provide their social media data will be contacted at baseline, months 3 and 6 to ask them to upload data. Participants will be given detailed guidance on how to request their data from the platforms and upload it to the study website via a unique link. Participants will receive a £10 voucher as a thank you for enrolling in the study, followed by £5 per month of continued participation and an additional £10 voucher for uploading their social media data at month 6.

**Table 2:** Outline of data collection measures over the 6-month follow-up period related to primary objectives

Measure	Description	Data collected at:						
		Baseline	Month 1	Month 2	Month 3	Month 4	Month 5	Month 6
Socio-demographic questionnaire	Including age, gender, ethnicity, education and/or employment status, smoking, alcohol consumption and drug use.	X						
Child and Adolescent Self-harm in Europe (CASE) study criteria^48^	Occurrence and characteristics of historical and current (ie, in the last month) self-harm events will be collected using an adapted self-report version of the two-item CASE study criteria.^48^	X	X	X	X	X	X	X
Self-reported social media use	Including which social media platforms are used, types of activity (eg, to share something, or to comment on a post) and average duration of daily usage.	X						X
Self-reported smartphone use	Including most frequently used apps, average duration of daily usage, and night-time access to a smartphone.	X						X
Smartphone Addiction Scale - Short Version (SAS-SV)^49^	Smartphone addiction will also be measured using the 10-item SAS-SV.^49^ Scores range between 10 and 60, with 31 and 33 representing a cut-off for smartphone addiction in adolescent boys and girls, respectively. The SAS-SV is a widely used and validated instrument which operationalises problematic smartphone usage.^50^	X				X		X
Social media data upload	Retrospective (ie, since the account was created) and prospective social media data will be uploaded by participants from their accounts on Facebook, Instagram, Snapchat, TikTok, Twitter and YouTube, including duration of usage (hours/min per day), timing of usage (diurnal/nocturnal posting), frequency of usage (visits to platforms per day), type of use and textual and imagery data.	X			X			X
Smartphone metadata extraction	Smartphone metadata will be extracted continuously for the duration of follow-up using the study software application, including intensity of usage by time of day, frequency and duration of incoming and outgoing telephone calls, frequency of incoming and outgoing text messages and length of time spent on software applications. For participants with Android phones, permissions will be set to enable the Android App Usage API to track daily statistics listed above. For iOS, low level system logs will be used to retrieve similar information as listed above, where iOS allows.	X	X	X	X	X	X	X
Electronic health records data extraction	Clinician recorded self-harm events, using a similar approach to Polling *et al,* 51 and other relevant clinical variables will be extracted from structured and free-text fields in EHR using the CRIS system. For participants who provide social media data, data will be extracted from the date of the first interaction on social media (or the date of the 13th birthday, whichever is later) to the end of the follow-up. For participants who do not provide social media data, data will be extracted from the date of the baseline assessment.	X						X

CRIS, Clinical Research Interactive Search; EHR, electronic health record.

**Table 3:** Outline of data collection measures over the 6-month follow-up period related to secondary objectives and other measurements

Measure	Description	Data collected at:						
		Baseline	Month 1	Month 2	Month 3	Month 4	Month 5	Month 6
Generalised Anxiety Disorder (GAD-7)^52^	Symptoms of anxiety will be measured using the 7-item GAD-7 scale.^52^ Scores range between 0 and 21, with scores of 5, 10 and 15 representing the cut-off points for mild, moderate and severe anxiety, respectively.	X				X		X
Patient Health Questionnaire (PHQ-9)^53^	Symptoms of depression will be measured using the 9-item PHQ-9.^53^ Scores range between 0 and 27, with scores of 5, 10, 15 and 20 representing the cut-off points for mild, moderate, moderately severe and severe depression, respectively.	X	X		X		X	X
PROMIS Sleep Disturbance Short Form / Paediatric Sleep Disturbance Short Form^54 55^	Symptoms of sleep disturbance will be measured using the 4-item Patient-Reported Outcomes Measurement Information System (PROMIS) Sleep Disturbance Short Form V.1.0 4a (for >18 year olds)^54^ or the Paediatric Sleep Disturbance Short Form V.1.0 4 a (for 5–17 year olds).^55^ Raw scores range between 4 and 16, and these are standardised using a T-score metric, with T-scores of 55, 60 and 70 representing the cut-off points for mild, moderate and severe sleep disturbance, respectively.	X		X		X		X
Three-Item Loneliness Scale^56^	Feelings of loneliness will be measured using the 3-item Loneliness Scale.^56^ Scores range between 3 and 9, with higher scores indicative of greater feelings of loneliness.	X			X			X
Eight-item bullying checklist^57^	Experiences of bullying victimisation will be measured using the 8-item bullying checklist^57^ derived from the Revised Olweus Bully/Victim Questionnaire.^58^ Eight statements are rated using a 5-point response scale, with ‘2 or 3 times a month’ representing a cut-off point for regular bullying victimisation.	X		X			X	X
Exposure to COVID-19 questionnaire*	Self-reported exposure to COVID-19 will be measured using two items from the Wave 2 survey of the Millennium Cohort Study.^59^	X						X

*Indicates that these are other measurements, not related to the primary and secondary objectives.

### Outcomes, exposures and other measurements

#### Primary outcome

Participant and clinician recorded self-harm events.

#### Secondary outcomes

Self-reported symptoms of anxiety, depression, sleep disturbance, loneliness and experiences of bullying victimisation.

#### Exposures

Social media metadata, textual and imagery data and smartphone metadata.

Self-reported social media and smartphone usage.

#### Other measurements

Self-reported exposure to COVID-19, sociodemographic and clinical covariates and process evaluation data.

### Data analysis plan

#### Descriptive analysis

Individuals will be grouped into those that have had an episode of self-harm, and those that have not. Frequency distributions for different types of self-harm will be summarised, and individuals with multiple, versus a single episode of self-harm will be compared. Baseline sociodemographic and clinical descriptors will be presented for those that self-harm and those that do not. Patterns of social media and smartphone use will be explored using a mixed methods analysis and presented as a descriptive analysis.

#### Primary analysis

Patterns of social media and smartphone use will be identified and categorised into specific problematic exposure groups. This will be defined using a mixed methods analysis comparing social media and smartphone data in the 7 days leading up to a self-harm episode. Individuals who do not experience an episode of self-harm will be randomly matched with four cases, and the same relative date and time of exposure will be randomly matched.

Social media and smartphone use will be compared between self-harm cases and non-cases, including social media platform of use, duration of use, time of use and absence of engagement (reduced level of posting but passive presence online). For example, night-time use compared with similar use during the day will be compared by self-harm status.

A binary generalised linear model with a log link will be fitted to derive the relative risk of a prospective self-harm episode in relation to a characteristic pattern of social media and smartphone use in the 7 days prior to the episode. Known social and physical determinants of health associated with self-harm will be included to adjust for baseline effects. Missing data will be explored for patterns of missingness. Secondary analyses will explore potential mediating effects of anxiety, depression, sleep disturbance, loneliness and bullying.

The primary analysis population under investigation will include all participants with a post-baseline assessment of self-harm.

#### Natural language processing and language analysis

In previous work by coauthor Liakata’s team^30^ aggregates of both language and smartphone features were obtained at hourly intervals preceding an episode (administration of a mood form). These were used in linear and non-linear regression models for prediction of mood scores. Recent work by Liakata’s team has developed natural language processing methods to identify changes in the mood of individuals over time through sequential analysis of 2-week user timelines on social media,^31^ and has shown that capturing such changes can be predictive of suicidality risk.^32^ The current protocol focuses on the 7-day window preceding a self-harm episode, making the above-mentioned research directly applicable to this work. While language features used in earlier work includes word embeddings (sentiment specific and others), n-grams, topics and lexica for sentiment, recent developments have shown the success of pretrained language models (eg, BERT from Devlin *et al*)^33^ in capturing information on mental health.^31 34^ The textual content of individuals posts will be represented using pretrained language models, combined with other smartphone data, to predict both moments of change and episodes of self-harm. Our earlier work on fusing heterogeneous features using multiple kernel learning,^30^ will provide useful baselines. Measurements of affective change both through our models for moments of change^31^ as well as more traditional measures of valence and affect^35^ will feed into the predictive models as well as the descriptive analysis. Models will be evaluated under robust cross validation settings to simulate as much as possible a real-world scenario and avoid overfitting.^36^

#### Qualitative analysis

Thematic analysis,^37^ which has been successfully applied in the analysis of social media data of patients and self-harm online communities,^38 39^ will be employed to explore characteristic patterns of social media use in the 7 days leading up to a self-harm episode from a qualitative perspective, complimentary to the main analyses. Thematic analysis comprises six stages: (1) familiarisation with the data; (2) generation of initial codes; (3) development of themes; (4) review of themes; and (5) defining themes; and (6) writing up the findings.^37^

Data from a purposive sample of cases will be selected for this analysis, based on age, sex, ethnicity, frequency of social media usage around the time of a self-harm episode and type of self-harm behaviour. Consistent with related work in this field (eg, Williams *et al*)^38 39^ around 500–600 social media postings will be analysed. Following removal of any personally identifiable/geographical material and duplicate material (for example, retweets on Twitter), the textual and imagery data of social media posts in the 7 days leading up to and including an episode of self-harm will be analysed using inductive and deductive methods to validate and refine the emerging themes.^40^ Inductive methods comprise repeated reading of the data to identify salient themes and deductive methods involve testing out predefined themes based on the research question and existing theories, for example, cry of pain theory/integrated motivational-volitional model.^41 42^ Analysis of visual data will include descriptive summaries of the visual content as well as analysis of thematic content. Coding frames for the analysis of textual and visual data will be developed by the study team, informed by relevant published literature (eg, Shanahan *et al*)^39^ the research question and predefined themes. The coding frames will be refined deductively throughout the analytical process.

### Process evaluation

Data from participant contact will be collected by the study team to evaluate the study processes for acceptability, feasibility and value. Reasons for decline and withdrawal will be coded and categorised where appropriate and summarised using descriptive statistics. A purposive sample of up to 20 participants (who provided consent to be contacted for further research when they enrolled in the 3S-YP study) will be invited to take part in a brief telephone or in person interview. Participants will complete a separate consent form and interviews will take place following study completion or drop out from the study (including participants who withdraw or stop providing data to the study). Interviews will be audio recorded and transcribed verbatim. Participants will receive a £20 voucher as a thank you for taking part. Interview data will be analysed using Braun and Clark’s^37^ thematic analysis approach, as outlined above.

## Ethics and dissemination

### Ethics and safety considerations

This study is being conducted in compliance with the principles of the Declaration of Helsinki^43^ and Good Clinical Practice as outlined in the UK Policy Framework for Health and Social Care Research.^44^

This study was approved by the National Research Ethics Service, London – Riverside (ethics ref 20/LO/1187; IRAS ref 269104), as well as by the Joint Research and Development Office of the Institute of Psychiatry, Psychology and Neuroscience and SLaM and the SLaM CRIS Oversight Committee (ref 20–074 and 21–039). The SLaM C4C patient research participation register was approved by the National Research Ethics Service, London – South East (ref 10/H0807/88), as well as by the SLaM Caldicott Guardian and the SLaM Trust Executive. The CRIS system was approved as a data set for secondary data analysis by the National Research Ethics Service, South Central – Oxford C (08/H06060/71). This study is registered on ClinicalTrials.gov.

Given the observational nature of the study, participation in this study is not anticipated to contribute to any specific adverse events. Drawing on our previous research and through careful planning with youth experts by experience, we have developed detailed procedures for conducting the study in a manner that is sensitive to the needs of young people, while maintaining high scientific standards and addressing any arising risks. Young people will provide their informed consent (or assent for young people aged 13–15 years old, along with consent from an adult with parental responsibility) prior to recruitment to the study. Participants can choose what data they want to share. Participants will be informed of the limits of confidentiality and their right to withdraw. Participants will be able to skip questions, take a break or terminate a questionnaire at any time. Participants will be provided with details of resources for accessing support and the study team’s contact information in the event of problems with any aspect of participation. The study team will have received training in the principles of Good Clinical Practice, informed consent, identifying and managing distress and risk (along with receiving regular supervision from the study chief investigator, RD) and the technical aspects of participation.

Data will be handled in accordance with the General Medical Council guidance on confidentiality,^45^ the principles of Good Clinical Practice, European General Data Protection Regulations^46^ and UK Data Protection Act.^47^

### Dissemination plan

Data collected in the study will not be made publicly available as it is not possible to guarantee that an individual could not be identified as a participant from their publicly available social media data. The findings will be disseminated through peer-reviewed scientific journals, conferences, websites, social media and stakeholder engagement activities.

### Project oversight

Project oversight is provided by our steering group, comprised of researchers and clinicians from across the fields of self-harm and suicide prevention, Child and Adolescent Mental Health Services(CAMHS), data science, public health, sleep medicine, cyberbullying and addictions, as well as young people with lived experience, representatives from the leading national UK youth mental health charity—YoungMinds, and communications and funding advisors. The group will meet at regular intervals throughout the study. The role of the group is to monitor study conduct and progress and contribute to decision-making and solution generation. The group will not have access to participants’ personal data.

### Patient and public involvement

Coauthor (SB-F), a senior service user consultant, and representatives from YoungMinds are facilitating an embedded participatory research approach to promote youth engagement and representation. Together we have developed safe working practices to ensure young people with lived experience are supported and able to contribute to the various engagement activities. Research priorities and consideration of key ethical issues were identified at the prefunding stage, through a series of consultations and workshops with youth service users from SLaM. When the study was being set-up, with support from YoungMinds, we partnered with youth advisors who joined the steering group and are contributing to co-production for the study duration. Study procedures and participant-facing digital tools and documentation were codesigned through consultations with the youth advisors and a workshop facilitated by YoungMinds with other young people to ensure the procedures and materials are acceptable and inclusive from a range of perspectives. Towards the end of data collection, we will conduct further engagement activities with YoungMinds to inform the analysis and dissemination plans.

### Study status

The study commenced recruitment in June 2021 and aims to conclude data collection in 2023.

## Supplementary Material

Reviewer comments

Author's manuscript
