# Supplementary material for: Observational prospective study of social media, smartphone use and self-harm in a clinical sample of young people: study protocol
Source: BMJ Open. 2023 Feb 1;13(2):e069748. doi: 10.1136/bmjopen-2022-069748 (PMC9896249; doi:10.1136/bmjopen-2022-069748)
Supplement: Author's manuscript [file bmjopen-2022-069748.draft_revisions.pdf]

BMJ Open

BMJ Open is committed to open peer review. As part of this commitment we make the peer review history of every article we publish publicly available.

When an article is published we post the peer reviewers' comments and the authors' responses online. We also post the versions of the paper that were used during peer review. These are the versions that the peer review comments apply to.

The versions of the paper that follow are the versions that were submitted during the peer review process. They are not the versions of record or the final published versions. They should not be cited or distributed as the published version of this manuscript.

BMJ Open is an open access journal and the full, final, typeset and author-corrected version of record of the manuscript is available on our site with no access controls, subscription charges or pay-per-view fees (http://bmjopen.bmj.com).

If you have any questions on BMJ Open's open peer review process please email info.bmjopen@bmj.com

BMJ Open

Observational prospective study of social media, smartphone use and self-harm in a clinical sample of young people: Study protocol

Journal:	*BMJ Open*
Manuscript ID	bmjopen-2022-069748
Article Type:	Protocol
Date Submitted by the Author:	01-Nov-2022

Complete List of Authors:	Bye, Amanda; King's College London Institute of Psychiatry Psychology and Neuroscience, Psychological Medicine<br>Carter, Ben; King's College London Institute of Psychiatry Psychology and Neuroscience, Biostatistics & Health Informatics<br>Leightley, Daniel; King's College London Institute of Psychiatry Psychology and Neuroscience, Psychological Medicine; King's College London Institute of Psychiatry Psychology and Neuroscience, King's Centre for Military Health Research<br>Trevillion, Kylee; King's College London Institute of Psychiatry Psychology and Neuroscience, Health Service and Population Research<br>Liakata, Maria; Queen Mary University of London, School of Electronic Engineering & Computer Science; The Alan Turing Institute<br>Branthonne-Foster , Stella; No affiliations<br>Williamson, Grace; King's College London Institute of Psychiatry Psychology and Neuroscience, Psychological Medicine<br>Zenasni, Zohra; King's College London Institute of Psychiatry Psychology and Neuroscience, Biostatistics and Health Informatics<br>Dutta, Rina; King's College London Institute of Psychiatry Psychology and Neuroscience, Psychological Medicine; South London and Maudsley NHS Foundation Trust

Keywords:	Suicide & self-harm < PSYCHIATRY, Child & adolescent psychiatry < PSYCHIATRY, MENTAL HEALTH, STATISTICS & RESEARCH METHODS, QUALITATIVE RESEARCH

SCHOLARONE™ Manuscripts

TITLE

Observational prospective study of social media, smartphone use and self-harm in a clinical sample of young people: Study protocol

AUTHORS

Dr Amanda Bye (0000-0002-9808-4956)

Research Associate

Department of Psychological Medicine, Institute of Psychiatry, Psychology and Neuroscience, King's College London, London, UK

Dr Ben Carter (0000-0003-0318-8865)

Reader in Medical Statistics

Department of Biostatistics and Health Informatics, Institute of Psychiatry, Psychology and Neuroscience, King's College London, London, UK

Dr Daniel Leightley (0000-0001-9512-752X)

Research Fellow

Department of Psychological Medicine, Institute of Psychiatry, Psychology and Neuroscience, King's College London, London, UK

King's Centre for Military Health Research, Institute of Psychiatry, Psychology and Neuroscience, King's College London, London, UK

Dr Kylee Trevillion (0000-0001-5865-4726)

Senior Lecturer

Health Service and Population Research Department, Institute of Psychiatry, Psychology and Neuroscience, King's College London, London, UK

Professor Maria Liakata (0000-0001-5765-0416)

Professor in Natural Language Processing

School of Electronic Engineering & Computer Science, Queen Mary, University of London, London, UK.

The Alan Turing Institute, London, UK.

University of Warwick, UK

For peer review only - http://bmjopen.bmj.com/site/about/guidelines.xhtml

Ms Stella Branthonne-Foster (0000-0002-5545-9199)

Lived experience advisor

London, UK

Ms Grace Williamson (0000-0002-4618-5819)

Research Assistant

Department of Psychological Medicine, Institute of Psychiatry, Psychology and Neuroscience, King's College London, London, UK

Ms Zohra Zenasni (0000-0002-2335-1378)

Research Associate

Department of Biostatistics and Health Informatics, Institute of Psychiatry, Psychology and Neuroscience, King's College London, London, UK.

Dr Rina Dutta (0000-0002-5614-8659)

Reader in Suicidology and Psychiatry / Consultant Psychiatrist

Department of Psychological Medicine, Institute of Psychiatry, Psychology and Neuroscience, King's College London, London, UK

South London and Maudsley NHS Foundation Trust, London, UK

CORRESPONDENCE

Dr Rina Dutta

Department of Psychological Medicine, Institute of Psychiatry, Psychology and Neuroscience, King's College London, London, UK

rina.dutta@kcl.ac.uk

WORD COUNT

3,857 words

For peer review only

For peer review only - http://bmjopen.bmj.com/site/about/guidelines.xhtml

ABSTRACT

Introduction

Young people are the most frequent users of social media and smartphones and there has been increasing speculation about the potential negative impacts of their use on mental health. This has coincided with a sharp increase in the levels of self-harm in young people. To date, studies researching this potential association are predominantly cross-sectional and reliant on self-report data, which precludes the ability to objectively analyse behaviour over time. This study is one of the first attempts to explore temporal patterns of real-world usage prior to self-harm, to identify whether there are usage patterns associated with an increased risk.

Methods and analysis

To study the mechanisms by which social media and smartphone use underpin self-harm in a clinical sample of young people, the Social media, Smartphone use and Self-harm in Young People (3S-YP) study uses a prospective, observational study design. Up to 600 young people aged 13-25 years old from secondary mental health services will be recruited and followed for up to 6 months. Primary analysis will compare real-world data in the seven days leading up to a participant or clinician recorded self-harm episode, to categorise patterns of problematic usage. Secondary analyses will explore potential mediating effects of anxiety, depression, sleep disturbance, loneliness and bullying.

Ethics and dissemination

This study was approved by the National Research Ethics Service, London - Riverside, as well as by the Joint Research and Development Office of the Institute of Psychiatry, Psychology and Neuroscience and South London and Maudsley NHS Foundation Trust (SLaM), and the SLaM Clinical Research Interactive Search (CRIS) Oversight Committee. The findings from this study will be disseminated through peer-reviewed scientific journals, conferences, websites, social media and stakeholder engagement activities.

Registration details

This study is registered on ClinicalTrials.gov (ID no. NCT04601220). Pre-results.

Protocol version

Version 1.4, 21/07/2022

Keywords

For peer review only

For peer review only - http://bmjopen.bmj.com/site/about/guidelines.xhtml

Suicide & self-harm; child & adolescent psychiatry; mental health; statistics & research methods; qualitative research; social media

STRENGTHS AND LIMITATIONS

- The Social media, Smartphone use and Self-harm in Young People (3S-YP) study uses a novel prospective, observational strategy to investigate the patterns of real-world social media and smartphone usage prior to episodes of self-harm in a clinical youth sample.
- Co-production with youth experts by experience, including co-author (SBF), a leading national UK youth mental health charity – YoungMinds, and a steering group comprising of experts in the field of self-harm and suicide prevention will ensure acceptability and relevance, as well as facilitate dissemination and impact.
- Due to the online nature of the study, participants need to be able to independently engage with the digital tools and study documentation that is only available in the English language.
- The flexible approach to participation regarding which data young people choose to share, will ensure our methods are acceptable and promote autonomy among participants, though it will increase the risk of missing and inconsistent data which could compromise the validity of the results and interpretations made.
- Mixed model analyses, state-of-the-art natural language processing methods combined with thematic analysis, will be used to analyse social media metadata, textual and imagery data and smartphone metadata.

For peer review only

INTRODUCTION

Self-harm is characterised as any behaviour where an individual causes damage or injury to their body in response to distress. Traditionally, research has focused more on severe cases of self-harm, i.e. cutting or self-poisoning, rather than a broader spectrum of behaviour, including burning, scratching, or hitting oneself. Self-harm in young people is a significant public health concern, with findings indicating that more than 15% of young people report engaging in self-harm in the community, coupled with high rates of repetition and elevated risk of suicide.[1–3] Prevalence figures are likely to underestimate the true scale of the problem, given young people are often reluctant to disclose and seek professional help owing to fear of stigma.[4] Furthermore, epidemiological studies tend to rely on hospital presentations and only reflect those cases of self-harm that are brought to medical attention.[5] Whereas community and school-based studies collect self-reported retrospective data. Therefore, whilst capturing a wider range of self-harm behaviours, they lack information about the specific timing and nature of non-help seeking self-harm.

Reasons for self-harm are complex and multifaceted, however there has been increasing speculation about the potential negative impacts of social media and smartphone use on youth mental health.[6] Young people are the most frequent users of social media and smartphones, with 87% of 12-15 year olds using social media and 95% of 13 year olds in the UK owning a smartphone.[7] Over the last decade, social media and smartphones have altered how young people spend their time and interact socially with peers.[8] This coincides with a sharp increase in the levels of self-harm, particularly amongst adolescent females.[9] Given the widespread use of social media and smartphones, understanding the implications for youth mental health is of paramount importance.

Social media platforms are often used by people with mental health problems to share personal experiences and seek support.[10] Feedback received can be perceived as supportive by the individual.[11] Thus, social media use can alleviate loneliness and social isolation that is common amongst young people with mental health problems as it can provide opportunities for strengthening existing friendships and developing new social connections, however, it can also exacerbate loneliness if excessive time is spent online rather than time that would be otherwise spent interacting socially offline.[12,13]

Online forums have been a cause for particular concern as users may be exposed to harmful self-harm content and online discussions that normalise behaviours and discourage help-seeking, which may be associated with increased suicidal ideation found among forum users compared to other forms of

For peer review only

For peer review only - http://bmjopen.bmj.com/site/about/guidelines.xhtml

social media user.[10] Other studies have found significant associations between cyberbullying victimisation and self-harm in young people, and to a lesser extent, cyberbullying perpetration, likely perpetuated by the anonymous nature of social media.[14] Young people engaging in self-harm behaviours may also be more likely to engage in risky online behaviour compared to their peers.[15] More recent findings indicate that while for some young people and in certain circumstances, social media can have a positive or neutral effect, for others it can be harmful and so more research is needed to explore the effects of different types of social media use on an individual level.[16,17]

Research on the effects on mental health of excessive screen time in general is more conflicting, possibly relating to inconsistencies in measurements and definitions employed.[18–20] Sohn *et al.*,[20] found evidence of an association between problematic smartphone usage and increased anxiety and depression. Emerging evidence indicates that problematic smartphone usage may be positively linked with suicide ideation and the association may be mediated by emotional intelligence.[21,22] Furthermore, there is an established literature linking excessive smartphone use and sleep disturbance[23] and poor sleep has been linked to depression and suicidal ideation in young people,[24] as well as there being an association between sleep problems and suicidal thoughts and behaviours independent of depression.[25]

Much of the current evidence on the association between social media and smartphone use and mental health and self-harm amongst young people is limited to associations from cross-sectional studies (e.g. Mancinelli *et al.*, 2021).[26] Where longitudinal studies are reported, they are based on small samples, are reliant on self-report data and have prolonged periods of time between waves of data collection (e.g. Arendt *et al.*, 2019).[27] These approaches have precluded the ability to explore potential mechanisms underlying self-harm behaviour over time using real-world data, in a prospective way. This study is one of the first to attempt to explore the temporal patterns of social media and smartphone use prior to episodes of self- and clinician reported self-harm events, to identify whether there are usage patterns of social media and smartphone use that are associated with increased risk of self-harm. The findings from this study have the potential for generating wide-ranging impact as well as filling the gaps in current knowledge. The overarching aim of the 3S-YP study is to investigate the mechanisms by which social media and smartphone use underpin self-harm in a clinical sample of young people. Primary objectives are:

- To identify characteristic patterns of social media and smartphone use in the seven days preceding an episode of self-harm.

For peer review only - http://bmjopen.bmj.com/site/about/guidelines.xhtml

- To determine whether characteristic patterns of social media and smartphone use associated with an episode of self-harm, differ from young people who have not had an episode of self-harm.

Secondary objectives are to investigate whether the association is mediated by sleep disturbance, depression, anxiety, loneliness, and bullying.

METHODS AND ANALYSIS

Study design

This study is a prospective six month cohort study and has been reported according to the STROBE Checklist for Cohort Studies (see Additional file 1) (NCT04601220, protocol version 1.4, 21/07/2022).

Study setting

Young people (aged 13 to 25 years) will be recruited from South London and Maudsley NHS Foundation Trust (SLaM). SLaM is one of the largest mental healthcare providers in Europe, providing secondary mental health care to a local population of approximately 1.3 million residents as well as national and specialist services.

Participant selection

Young people will be identified via one of the following selection methods:

1) SLaM's Consent for Contact (C4C) patient research participation register of patients who have given prior consent to researchers with ethically approved studies to access their health records and contact them for the purposes of recruitment to research for which they are eligible.[28] A researcher who is independent of the study team and who works for the NIHR Biomedical Research Centre (BRC) within SLaM, will apply the eligibility criteria to the register using the Clinical Research Interactive Search (CRIS) system to screen for potentially eligible young people. The CRIS system is a case register that contains de-identified information extracted from the Trust's Electronic Health Record (EHR) system.[29] The study team will then be supplied with the Trust-identifying details of potentially eligible young people so they can check their EHRs in detail for clinical eligibility. For eligible young people, the study team will inform the care coordinator of the intention to approach prior to initiating any contact. This is so that care coordinators can advise if it is not appropriate for a young person under their care to be approached for the study.
2) If the above yields insufficient numbers, we will invite clinicians to refer young people under their care directly to the study team. We will attend team meetings to explain the study and

For peer review only - http://bmjopen.bmj.com/site/about/guidelines.xhtml

eligibility criteria to clinicians. Clinicians will screen young people under their care for eligibility and will contact eligible young people (and a parent or carer with parental responsibility for young people aged 13-15 years old) to explain the study and seek permission to share their contact details with the study team.

Eligibility criteria

Inclusion and exclusion criteria are reported in Table 1. No recruitment quotas will be imposed, with respect to any participant characteristics. To reduce the risk of participant selection bias, all eligible young people will be invited to participate until the recruitment target is met.

Table 1: Eligibility criteria

Inclusion criteria	• Identified via SLaM's C4C patient research participation register or referral to the study team by their clinician.<br>• Aged 13-25 years old at the time of study approach.<br>• Accessed mental health services at SLaM in the last 12 months.<br>• Has capacity to consent (and an adult with parental responsibility for young people aged 13-15 years old). Mental capacity will be assumed unless evidence from a clinician or during contact with the study team suggests otherwise.
Exclusion criteria	• Unable to complete the questionnaires via the study software application or online survey platform.<br>• Admitted to an inpatient psychiatric ward, sectioned under the Mental Health Act or in prison at the time of approach.<br>• Clinician advises it is not appropriate to approach.

Sample size calculation

In a recent national community survey, the reported annual age-sex incidence of self-harm for young people aged 16-18 years old was 36.4% (95%CI 22.4–52.2) for males and 43.5% (95%CI 35.5–51.7) for females.[3] Based on uptake of SLaM C4C patient research participation register, we estimate that if 600 young people consent to take part, 480 will be followed up during a 6-month period, resulting in at least 150 with an episode of self-harm. Using the most conservative estimates, if approximately 22.5% of those who do not display characteristic problematic patterns of social media usage (or smartphone usage) have an episode of self-harm and this increases to 35% in those identified with

For peer review only - http://bmjopen.bmj.com/site/about/guidelines.xhtml

problematic usage in the previous week, we expect 86% power to detect a 12.5% increase in self-harm at 5% significance, assuming 480 young people are followed up.

Recruitment method and study procedures

The study team will contact eligible young people (and/or an adult with parental responsibility for young people aged 13-15 years old) by telephone, text or email to invite the young person to take part. They will be sent a unique link to the study website which will host the participant information and online consent and assent forms as well as being offered the option of a face-to-face or virtual meeting if needed. Young people aged 16-25 years old who want to take part, will be asked to confirm their consent via an online form. This will include separate consent for the following types of data collection: questionnaires, EHRs, social media and smartphone data; choosing not to consent to the latter three will not preclude participation. For young people aged 13-15 years old, an adult with parental responsibility will first be asked to confirm their consent via an online form, following which the young person will be asked to confirm their assent. A maximum of three contact attempts will be made to non-responders. The flow of participants through the study will be documented in a flowchart (see Figure 1 - Participant flowchart).

Participants will then install the study software application on their smartphone device and complete the baseline questionnaires. The study software application has been designed to continuously extract smartphone metadata and to deliver the questionnaires for the follow-up period (up to 6 months, reduced from 12 months following an initial pilot phase). Tables 2 and 3 outlines data collection measures over the 6 month follow-up period related to primary and secondary objectives and other measurements. Participants who choose not to provide smartphone metadata (or use the study software application for any other reason) will be provided with a unique link to an online survey platform – Qualtrics (https://www.qualtrics.com) – to complete the questionnaires according to the same schedule. Participants will receive a reminder (an automated push notification and/or a pre-scheduled standardised text message and/or email) on the day a questionnaire is due, followed by a second reminder seven days later if incomplete. The final set of questionnaires may also be sent via post. Participants who consent to provide their social media data will be contacted at baseline, months 3 and 6 to ask them to upload data. Participants will be given detailed guidance on how to request their data from the platforms and upload it to the study website via a unique link. Participants will receive a £10 voucher as a thank you for enrolling in the study, followed by £5 per month of continued participation and an additional £10 voucher for uploading their social media data at month 6.

INSERT FIGURE 1 HERE

For peer review only - http://bmjopen.bmj.com/site/about/guidelines.xhtml

Table 2: Outline of data collection measures over the 6 month follow-up period related to primary objectives

Measure	Description	Data collected at:						
		Baseline	Month 1	Month 2	Month 3	Month 4	Month 5	Month 6
Socio-demographic questionnaire	Including age, gender, ethnicity, education and/or employment status, smoking, alcohol consumption and drug use.	X						
Child and Adolescent Self-harm in Europe (CASE) Study criteria [30]	Occurrence and characteristics of historical and current (i.e. in the last month) self-harm events will be collected using an adapted self-report version of the 2-item CASE Study criteria.[30]	X	X	X	X	X	X	X
Self-reported social media use	Including which social media platforms are used, types of activity (e.g. to share something, or to comment on a post), and average duration of daily usage.	X						X
Self-reported smartphone use	Including most frequently used apps, average duration of daily usage, and night-time access to smartphone.	X						X
Smartphone Addiction Scale - Short Version (SAS-SV) [38]	Smartphone addiction will also be measured using the 10-item Smartphone Addiction Scale-Short Version (SAS-SV).[SAS-SV; 38]<br>Scores range between 10 and 60, with 31 and 33 representing a cut-off for smartphone addiction in adolescent boys and girls, respectively. The SAS-SV is a widely used and validated instrument which operationalises problematic smartphone usage.[39]	X				X		X
Social media data upload	Retrospective (i.e. since the account was created) and prospective social media data will be uploaded by participants from their accounts on Facebook, Instagram, Snapchat, TikTok, Twitter and YouTube, including duration	X			X			X

For peer review only - http://bmjopen.bmj.com/site/about/guidelines.xhtml

	of usage (hours/minutes per day), timing of usage (diurnal/nocturnal posting), frequency of usage (visits to platforms per day), type of use, and textual and imagery data.							
Smartphone metadata extraction	Smartphone metadata will be extracted continuously for the duration of follow-up using the study software application, including intensity of usage by time of day, frequency and duration of incoming and outgoing telephone calls, frequency of incoming and outgoing text messages and length of time spent on software applications.<br>For participants with Android phones, permissions will be set to enable the Android App Usage API to track daily statistics listed above. For iOS, low level system logs will be used to retrieve similar information as listed above, where iOS allows.	X	X	X	X	X	X	X
Electronic health records data extraction	Clinician recorded self-harm events, using a similar approach to Polling *et al.*,[41] and other relevant clinical variables will be extracted from structured and free-text fields in EHR using the CRIS system.<br>For participants who provide social media data, data will be extracted from the date of the first interaction on social media (or the date of the thirteenth birthday, whichever is later) to the end of the follow up. For participants who do not provide social media data, data will be extracted from the date of the baseline assessment.	X						X

For peer review only

Table 3: Outline of data collection measures over the 6 month follow-up period related to secondary objectives and other measurements

Measure	Description	Data collected at:						
		Baseline	Month 1	Month 2	Month 3	Month 4	Month 5	Month 6
Generalised Anxiety Disorder (GAD-7) [31]	Symptoms of anxiety will be measured using the 7-item Generalized Anxiety Disorder Scale (GAD-7).[31] Scores range between 0 and 21, with scores of 5, 10, and 15 representing the cut-off points for mild, moderate, and severe anxiety, respectively.	X				X		X
Patient Heath Questionnaire (PHQ-9) [32]	Symptoms of depression will be measured using the 9-item Patient Health Questionnaire (PHQ-9).[32] Scores range between 0 and 27, with scores of 5, 10, 15, and 20 representing the cut-off points for mild, moderate, moderately severe, and severe depression, respectively.	X	X		X		X	X
PROMIS Sleep Disturbance Short Form / Pediatric Sleep Disturbance Short Form [33,34]	Symptoms of sleep disturbance will be measured using the 4-item Patient-Reported Outcomes Measurement Information System (PROMIS) Sleep Disturbance Short Form V1.0 4a (for >18 year olds) [33] or the Pediatric Sleep Disturbance Short Form V1.0 4a (for 5-17 year olds).[34] Raw scores range between 4 and 16, and these are standardized using a T-score metric, with T-scores of 55, 60 and 70 representing the cut-off points for mild, moderate, and severe sleep disturbance, respectively.	X		X		X		X
Three-Item Loneliness Scale [35]	Feelings of loneliness will be measured using the 3-item Loneliness Scale.[35] Scores range between 3 and 9, with higher scores indicative of greater feelings of loneliness.	X			X			X

For peer review only

For peer review only - http://bmjopen.bmj.com/site/about/guidelines.xhtml

Eight-item bullying checklist [36]	Experiences of bullying victimisation will be measured using the 8-item Bullying Checklist[36] derived from the Revised Olweus Bully/Victim Questionnaire (OBVQ-R).[37] Eight statements are rated using a 5-point response scale, with “2 or 3 times a month” representing a cut-off point for regular bullying victimisation.	X	X	X	X
Exposure to Covid-19 questionnaire*	Self-reported exposure to Covid-19 will be measured using 2 items from the Wave 2 survey of the Millennium Cohort Study (MCS).[40]	X			X

*Indicates that these are other measurements, not related to the primary and secondary objectives

For peer review only

For peer review only - http://bmjopen.bmj.com/site/about/guidelines.xhtml

Outcomes, exposures and other measurements

Primary outcome

Participant and clinician recorded self-harm events.

Secondary outcomes

Self-reported symptoms of anxiety, depression, sleep disturbance, loneliness and experiences of bullying victimisation.

Exposures

Social media metadata, textual and imagery data and smartphone metadata.

Self-reported social media and smartphone usage.

Other measurements

Self-reported exposure to Covid-19, sociodemographic and clinical covariates and process evaluation data.

Data analysis plan

Descriptive analysis

Individuals will be grouped into those that have had an episode of self-harm, and those that have not. Frequency distributions for different types of self-harm will be summarised, and individuals with multiple, versus a single episode of self-harm will be compared. Baseline sociodemographic and clinical descriptors will be presented for those that self-harm and those that do not. Patterns of social media and smartphone use will be explored using a mixed methods analysis and presented as a descriptive analysis.

Primary analysis

Patterns of social media and smartphone use will be identified and categorised into specific problematic exposure groups. This will be defined using a mixed methods analysis comparing social media and smartphone data in the seven days leading up to a self-harm episode. Individuals who do not experience an episode of self-harm will be randomly matched with four cases, and the same relative date and time of exposure will be randomly matched.

For peer review only - http://bmjopen.bmj.com/site/about/guidelines.xhtml

For peer review only

Social media and smartphone use will be compared between self-harm cases and non-cases, including social media platform of use, duration of use, time of use and absence of engagement (reduced level of posting but passive presence online). For example, night-time use compared to similar use during the day will be compared by self-harm status.

A binary generalized linear model with a log link will be fitted to derive the relative risk of a prospective self-harm episode in relation to a characteristic pattern of social media and smartphone use in the seven days prior to the episode. Known social and physical determinants of health associated with self-harm will be included to adjust for baseline effects. Missing data will be explored for patterns of missingness. Secondary analyses will explore potential mediating effects of anxiety, depression, sleep disturbance, loneliness and bullying.

The primary analysis population under investigation will include all participants with a post-baseline assessment of self-harm.

Natural Language Processing (NLP) and language analysis

In previous work by co-author Liakata's team[42] aggregates of both language and smartphone features were obtained at hourly intervals preceding an episode (administration of a mood form). These were used in linear and non-linear regression models for prediction of mood scores. Recent work by Liakata's team has developed NLP methods to identify changes in the mood of individuals over time through sequential analysis of two-week user timelines on social media,[43] and has shown that capturing such changes can be predictive of suicidality risk.[44] The current protocol focuses on the seven-day window preceding a self-harm episode, making the above-mentioned research directly applicable to this work. While language features used in earlier work includes word embeddings (sentiment specific and others), n-grams, topics, and lexica for sentiment, recent developments have shown the success of pre-trained language models (e.g. BERT from Devlin *et al.*, 2019)[45] in capturing information on mental health.[43,46] The textual content of individuals posts will be represented using pre-trained language models, combined with other smartphone data, to predict both moments of change and episodes of self-harm. Our earlier work on fusing heterogeneous features using multiple kernel learning,[42] will provide useful baselines. Measurements of affective change both through our models for moments of change[43] as well as more traditional measures of valence and affect[47] will feed into the predictive models as well as the descriptive analysis. Models will be evaluated under robust cross validation settings to simulate as much as possible a real-world scenario and avoid overfitting.[48]

For peer review only - http://bmjopen.bmj.com/site/about/guidelines.xhtml

Qualitative analysis

Thematic analysis,[49] which has been successfully applied in the analysis of social media data of patients and self-harm online communities,[50,51] will be employed to explore characteristic patterns of social media use in the seven days leading up to a self-harm episode from a qualitative perspective, complimentary to the main analyses. Thematic analysis comprises six stages: (1) familiarisation with the data; (2) generation of initial codes; (3) development of themes; (4) review of themes; and (5) defining themes; and (6) writing up the findings.[49]

Data from a purposive sample of cases will be selected for this analysis, based on age, sex, ethnicity, frequency of social media usage around the time of a self-harm episode and type of self-harm behaviour. Consistent with related work in this field (e.g. Shanahan *et al*., 2019; Williams *et al*., 2018)[50,51] around 500-600 social media postings will be analysed. Following removal of any personally identifiable/geographical material and duplicate material (for example, re-tweets on Twitter), the textual and imagery data of social media posts in the seven days leading up to and including an episode of self-harm will be analysed using inductive and deductive methods to validate and refine the emerging themes.[52] Inductive methods comprise repeated reading of the data to identify salient themes and deductive methods involve testing out pre-defined themes based on the research question and existing theories, for example, cry of pain theory/integrated motivational-volitional model.[53,54] Analysis of visual data will include descriptive summaries of the visual content as well as analysis of thematic content. Coding frames for the analysis of textual and visual data will be developed by the study team, informed by relevant published literature (e.g. Shanahan *et al*., 2019)[51] the research question and pre-defined themes. The coding frames will be refined deductively throughout the analytical process.

Process evaluation

Data from participant contact will be collected by the study team to evaluate the study processes for acceptability, feasibility and value. Reasons for decline and withdrawal will be coded and categorised where appropriate and summarised using descriptive statistics. A purposive sample of up to 20 participants (who provided consent to be contacted for further research when they enrolled in the 3S-YP study) will be invited to take part in a brief telephone or in person interview. Participants will complete a separate consent form and interviews will take place following study completion or drop out from the study (including participants who withdraw or stop providing data to the study). Interviews will be audio recorded and transcribed verbatim. Participants will receive a £20 voucher as

For peer review only - http://bmjopen.bmj.com/site/about/guidelines.xhtml

a thank you for taking part. Interview data will be analysed using Braun and Clark's[49] thematic analysis approach, as outlined above.

ETHICS AND DISSEMINATION

Ethics and safety considerations

This study is being conducted in compliance with the principles of the Declaration of Helsinki[55] and Good Clinical Practice as outlined in the UK Policy Framework for Health and Social Care Research.[56]

This study was approved by the National Research Ethics Service, London – Riverside (ethics ref 20/LO/1187; IRAS ref 269104), as well as by the Joint Research and Development Office of the Institute of Psychiatry, Psychology and Neuroscience and SLaM and the SLaM CRIS Oversight Committee (ref 20-074 and 21-039). The SLaM C4C patient research participation register was approved by the National Research Ethics Service, London – South East (ref 10/H0807/88), as well as by the SLaM Caldicott Guardian and the SLaM Trust Executive. The CRIS system was approved as a dataset for secondary data analysis by the National Research Ethics Service, South Central – Oxford C (08/H06060/71). This study is registered on ClinicalTrials.gov (ref NCT04601220).

Given the observational nature of the study, participation in this study is not anticipated to contribute to any specific adverse events. Drawing on our previous research and through careful planning with youth experts by experience, we have developed detailed procedures for conducting the study in a manner that is sensitive to the needs of young people, while maintaining high scientific standards and addressing any arising risks. Young people will provide their informed consent (or assent for young people aged 13-15 years old, along with consent from an adult with parental responsibility) prior to recruitment to the study. Participants can choose what data they want to share. Participants will be informed of the limits of confidentiality and their right to withdraw. Participants will be able to skip questions, take a break, or terminate a questionnaire at any time. Participants will be provided with details of resources for accessing support and the study team's contact information in the event of problems with any aspect of participation. The study team will have received training in the principles of Good Clinical Practice, informed consent, identifying and managing distress and risk (along with receiving regular supervision from the study chief investigator, RD), and the technical aspects of participation.

For peer review only

For peer review only - http://bmjopen.bmj.com/site/about/guidelines.xhtml

Data will be handled in accordance with the General Medical Council guidance on confidentiality,(2009) the principles of Good Clinical Practice, European General Data Protection Regulations[58] and UK Data Protection Act.[59]

Dissemination plan

Data collected in the study will not be made publicly available as it is not possible to guarantee that an individual could not be identified as a participant from their publicly available social media data. The findings will be disseminated through peer-reviewed scientific journals, conferences, websites, social media and stakeholder engagement activities.

PROJECT OVERSIGHT

Project oversight is provided by our steering group, comprised of researchers and clinicians from across the fields of self-harm and suicide prevention, CAMHS, data science, public health, sleep medicine, cyberbullying and addictions, as well as young people with lived experience, representatives from the leading national UK youth mental health charity – YoungMinds, and communications and funding advisors. The group will meet at regular intervals throughout the study. The role of the group is to monitor study conduct and progress and contribute to decision making and solution generation. The group will not have access to participants' personal data.

PATIENT AND PUBLIC INVOLVEMENT

Co-author (SBF), a senior service user consultant, and representatives from YoungMinds are facilitating an embedded participatory research approach to promote youth engagement and representation. Together we have developed safe working practices to ensure young people with lived experience are supported and able to contribute to the various engagement activities. Research priorities and consideration of key ethical issues were identified at the pre-funding stage, through a series of consultations and workshops with youth service users from SLaM. When the study was being set-up, with support from YoungMinds, we partnered with two youth advisors who joined the steering group and contribute to co-production for the study duration. Study procedures and participant-facing digital tools and documentation were co-designed through consultations with our youth advisors and a workshop facilitated by YoungMinds with other young people to ensure the procedures and materials are acceptable and inclusive from a range of perspectives. Towards the end of data collection, we will conduct further engagement activities with YoungMinds to inform the analysis and dissemination plans.

For peer review only

STUDY STATUS

The study commenced recruitment in June 2021 and aims to conclude data collection in 2023.

AVAILABILITY OF DATA AND MATERIALS

Not applicable – this manuscript does not contain any data.

ACKNOWLEDGEMENTS

We would like to thank all the members of the steering group, including Louise Arsenault, Janis Baird, Johnny Downs, Tamsin Ford, Nuala Flewett, Paul Gringras, Matthew Hotopf, Hazel Inskip, Nicola Kalk, Navneet Kapur, Fiona Lacey, Dennis Ougrin, Louise Pratt, Angus Roberts, Edmund Sonuga-Barke, Robert Stewart, Jack Stonebridge, Alastair Sutcliffe, Sumithra Velupillai and Tony Wood, and the young people who have been involved in co-production activities for their invaluable contributions to the development of the 3S-YP study. We would like to thank YoungMinds for partnering with us. We would also like to thank local NIHR CRN South London staff for support in recruiting participants.

AUTHOR CONTRIBUTIONS

RD, BC, KT, SBF and ML contributed to the conception of the study and obtaining the funding. RD is the study CI. RD, BC, KT, SBF, ML, DL and AB contributed to the design of the study. BC performed the power analysis. RD, BC, KT, SBF, ML, DL, AB and ZZ contributed to the analysis plans. DL, AB, SBF, RD and GW contributed to the development or testing of the systems and software application used for data collection and management. DL, AB and RD contributed to the development of the data protection and storage systems. AB prepared the protocol and other study documentation for ethical approval and the first draft of the manuscript for publication, and is responsible for the study management, with oversight by RD. All authors contributed to revising the manuscript and approved the final version to be published. All authors have agreed to be accountable for all aspects of the work in ensuring that questions related to the accuracy or integrity of any part of the work are appropriately investigated and resolved.

FUNDING STATEMENT

This paper represents independent research supported by the Medical Research Council and Medical Research Foundation (grant number MR/S020365/1). This research was also part supported by the National Institute for Health Research (NIHR) Biomedical Research Centre at South London and Maudsley NHS Foundation Trust and King's College London. RD was also funded by a Clinician Scientist Fellowship from the Health Foundation in partnership with the Academy of Medical Sciences and her

For peer review only - http://bmjopen.bmj.com/site/about/guidelines.xhtml

work is supported by the National Institute for Health Research (NIHR) Biomedical Research Centre at South London and Maudsley NHS Foundation Trust and King's College London. BC is also supported by the Nuffield Trust. ML is also supported by the Engineering and Physical Sciences Research Council (grant number EP/V030302/1) and The Alan Turing Institute (grant number EP/N510129/1). DL is also supported by the Forces in Mind Trust (Project number: FIMT/0323KCL), a funding scheme run by the Forces in Mind Trust using an endowment awarded by the National Lottery Community Fund. The views expressed are those of the author(s) and not necessarily those of the MRC, the MRF, the NHS, the NIHR or the Department of Health and Social Care.

COMPETING INTERESTS STATEMENT

None of the authors declares a conflict of interest.

PATIENT CONSENT REQUIRED FOR PUBLICATION

Not required.

REFERENCES

1 Hawton K, Bergen H, Kapur N, *et al.* Repetition of self-harm and suicide following self-harm in children and adolescents: findings from the Multicentre Study of Self-harm in England. *Journal of Child Psychology and Psychiatry* 2012;**53**:1212–9. doi:10.1111/J.1469-7610.2012.02559.X

2 Bennardi M, McMahon E, Corcoran P, *et al.* Risk of repeated self-harm and associated factors in children, adolescents and young adults. *BMC Psychiatry* 2016;**16**:1–12. doi:10.1186/s12888-016-1120-2

3 Morey Y, Mellon D, Dailami N, *et al.* Adolescent self-harm in the community: an update on prevalence using a self-report survey of adolescents aged 13-18 in England. *J Public Health (Oxf)* 2017;**39**:58–64. doi:10.1093/pubmed/fdw010

4 Rowe SL, French RS, Henderson C, *et al.* Help-seeking behaviour and adolescent self-harm: A systematic review. *Australian and New Zealand Journal of Psychiatry* 2014;**48**:1083–95. doi:10.1177/0004867414555718

5 Hawton K, Saunders KEA, O'Connor RC. Self-harm and suicide in adolescents. The Lancet. 2012;**379**:2373–82. doi:10.1016/S0140-6736(12)60322-5

6 Abi-Jaoude E, Naylor KT, Pignatiello A. Smartphones, social media use and youth mental health. *CMAJ* 2020;**192**:E136–41. doi:10.1503/CMAJ.190434

7 Ofcom. Children and parents: media use and attitudes report Content consumption and online activities. 2021. https://www.ofcom.org.uk/__data/assets/pdf_file/0025/217825/children-and-parents-media-use-and-attitudes-report-2020-21.pdf (accessed 14 Oct 2022).

For peer review only - http://bmjopen.bmj.com/site/about/guidelines.xhtml

8 Twenge JM, Spitzberg BH, Campbell WK. Less in-person social interaction with peers among U.S. adolescents in the 21st century and links to loneliness. *J Soc Pers Relat* 2019;**36**:1892–913. doi:10.1177/0265407519836170

9 Morgan C, Webb RT, Carr MJ, *et al.* Incidence, clinical management, and mortality risk following self harm among children and adolescents: cohort study in primary care. *BMJ* 2017;**359**:j4351. doi:10.1136/bmj.j4351

10 Daine K, Hawton K, Singaravelu V, *et al.* The Power of the Web: A Systematic Review of Studies of the Influence of the Internet on Self-Harm and Suicide in Young People. *PLoS One* 2013;**8**:e77555. doi:10.1371/journal.pone.0077555

11 de Choudhury M, De S. Mental Health Discourse on reddit: Self-Disclosure, Social Support, and Anonymity. *Proceedings of the Eighth International AAAI Conference on Weblogs and Social Media* 2014;**8**:71–80.

12 Nowland R, Necka EA, Cacioppo JT. Loneliness and Social Internet Use: Pathways to Reconnection in a Digital World? *https://doi.org/101177/1745691617713052* 2017;**13**:70–87. doi:10.1177/1745691617713052

13 Matthews T, Danese A, Caspi A, *et al.* Lonely young adults in modern Britain: findings from an epidemiological cohort study. *Psychol Med* 2019;**49**:268–77. doi:10.1017/S0033291718000788

14 John A, Glendenning AC, Marchant A, *et al.* Self-Harm, Suicidal Behaviours, and Cyberbullying in Children and Young People: Systematic Review. *J Med Internet Res* 2018;**20**. doi:10.2196/JMIR.9044

15 Mitchell KJ, Ybarra ML. Online behavior of youth who engage in self-harm provides clues for preventive intervention. *Prev Med (Baltim)* 2007;**45**:392–6. doi:10.1016/J.YPMED.2007.05.008

16 Beyens I, Loes Pouwels J, van Driel II, *et al.* Social Media Use and Adolescents' Well-Being: Developing a Typology of Person-Specific Effect Patterns. *Science Reports* 2020;**10**. doi:10.1177/00936502211038196

17 Sedgwick R, Epstein S, Dutta R, *et al.* Social media, internet use and suicide attempts in adolescents. *Curr Opin Psychiatry* 2019;**32**:534–41. doi:10.1097/YCO.0000000000000547

18 Orben A, Przybylski AK. The association between adolescent well-being and digital technology use. *Nat Hum Behav* 2019;**3**:173–82. doi:10.1038/S41562-018-0506-1

19 Orben A, Przybylski AK. Corrigendum: Screens, Teens, and Psychological Well-Being: Evidence From Three Time-Use-Diary Studies. *Psychol Sci* 2019;**30**:682–696. doi:10.1177/0956797619862548

20 Sohn S, Rees P, Wildridge B, *et al.* Prevalence of problematic smartphone usage and associated mental health outcomes amongst children and young people: a systematic review, meta-analysis and GRADE of the evidence. *BMC Psychiatry* 2019;**19**:356. doi:10.1186/s12888-019-2350-x

21 Arrivillaga C, Rey L, Extremera N. Adolescents' problematic internet and smartphone use is related to suicide ideation: Does emotional intelligence make a difference? *Comput Human Behav* 2020;**110**:106375. doi:10.1016/j.chb.2020.106375

For peer review only - http://bmjopen.bmj.com/site/about/guidelines.xhtml

22 Lung F-W, Shu B-C, Chiang T-L, *et al.* Relationships between internet use, deliberate self-harm, and happiness in adolescents: A Taiwan birth cohort pilot study. *PLoS One* 2020;**15**:e0235834. doi:10.1371/journal.pone.0235834

23 Carter B, Rees P, Hale L, *et al.* Association Between Portable Screen-Based Media Device Access or Use and Sleep Outcomes A Systematic Review and Meta-analysis. *JAMA Pediatr* 2016;**170**:1202–8. doi:10.1001/jamapediatrics.2016.2341

24 Gangwisch JE, Babiss LA, Malaspina D, *et al.* Earlier parental set bedtimes as a protective factor against depression and suicidal ideation. *Sleep* 2010;**33**:97–106. doi:10.1093/SLEEP/33.1.97

25 Pigeon WR, Pinquart M, Conner K. Meta-analysis of sleep disturbance and suicidal thoughts and behaviors. *J Clin Psychiatry* 2012;**73**. doi:10.4088/JCP.11R07586

26 Mancinelli E, Sharka O, Lai T, *et al.* Self-injury and Smartphone Addiction: Age and gender differences in a community sample of adolescents presenting self-injurious behavior. *Health Psychol Open* 2021;**8**. doi:10.1177/20551029211038811

27 Arendt F, Scherr S, Romer D. Effects of exposure to self-harm on social media: Evidence from a two-wave panel study among young adults. *New Media Soc* 2019;**21**:2422–42. doi:10.1177/1461444819850106

28 Callard F, Broadbent M, Denis M, *et al.* Developing a new model for patient recruitment in mental health services: a cohort study using Electronic Health Records. *BMJ Open* 2014;**4**:5654. doi:10.1136/bmjopen-2014-005654

29 Perera G, Broadbent M, Callard F, *et al.* Cohort profile of the South London and Maudsley NHS Foundation Trust Biomedical Research Centre (SLaM BRC) Case Register: current status and recent enhancement of an Electronic Mental Health Record-derived data resource. *BMJ Open* 2016;**6**:8721. doi:10.1136/bmjopen-2015

30 Madge N, Hewitt A, Hawton K, *et al.* Deliberate self-harm within an international community sample of young people: comparative findings from the Child & Adolescent Self-harm in Europe (CASE) Study. *Journal of Child Psychology and Psychiatry* 2008;**49**:667–77. doi:10.1111/j.1469-7610.2008.01879.x

31 Spitzer RL, Kroenke K, Williams JBW, *et al.* A Brief Measure for Assessing Generalized Anxiety Disorder. *Arch Intern Med* 2006;**166**:1092. doi:10.1001/archinte.166.10.1092

32 Kroenke K, Spitzer RL, Williams JBW. The PHQ-9: Validity of a brief depression severity measure. *J Gen Intern Med* 2001;**16**:606–13. doi:10.1046/j.1525-1497.2001.016009606.x

33 Yu L, Buysse DJ, Germain A, *et al.* Development of Short Forms From the PROMIS™ Sleep Disturbance and Sleep-Related Impairment Item Banks. *Behavioral Sleep Medicine* 2011;**10**:6–24. doi:10.1080/15402002.2012.636266

34 Forrest CB, Meltzer LJ, Marcus CL, *et al.* Development and validation of the PROMIS Pediatric Sleep Disturbance and Sleep-Related Impairment item banks. *Sleep* 2018;**41**:1–13. doi:10.1093/sleep/zsy054

35 Hughes ME, Waite LJ, Hawkley LC, *et al.* A Short Scale for Measuring Loneliness in Large Surveys: Results From Two Population-Based Studies. *Res Aging* 1999;**26**:655–72.

For peer review only - http://bmjopen.bmj.com/site/about/guidelines.xhtml

36 Przybylski AK, Bowes L. Cyberbullying and adolescent well-being in England: a population-based cross-sectional study. *Lancet Child Adolesc Health* 2017;**1**:19–26. doi:10.1016/S2352-4642(17)30011-1

37 Olweus D. Revised Olweus Bully/Victim Questionnaire. 1996;:0–12. doi:10.1037/t09634-000

38 Kwon M, Kim D-J, Cho H, *et al.* The Smartphone Addiction Scale: Development and Validation of a Short Version for Adolescents. *PLoS One* 2013;**8**:e83558. doi:10.1371/journal.pone.0083558

39 Olson JA, Sandra DA, Colucci ÉS, *et al.* Smartphone addiction is increasing across the world: A meta-analysis of 24 countries. *Comput Human Behav* 2022;**129**:107138. doi:10.1016/J.CHB.2021.107138

40 University College London UI of EC for LS. COVID-19 Survey in Five National Longitudinal Cohort Studies: Millennium Cohort Study, Next Steps, 1970 British Cohort Study and 1958 National Child Development Study, 2020-2021. 4th Edition. UK Data Service. SN: 8658. 2022.

41 Polling C, Tulloch A, Banerjee S, *et al.* Using routine clinical and administrative data to produce a dataset of attendances at Emergency Departments following self-harm. *BMC Emerg Med* 2015;**15**:1–8. doi:10.1186/S12873-015-0041-6/TABLES/4

42 Tsakalidis A, Liakata M, Damoulas T, *et al.* Combining Heterogeneous User Generated Data to Sense Well-being. In: *Proceedings of COLING 2016, the 26th International Conference on Computational Linguistics : Technical Papers*. 2016. 3007–18.http://wing.comp.nus.edu.sg:8080/SMSCorpus/contribution.jsp (accessed 5 Jun 2020).

43 Tsakalidis A, Nanni F, Hills A, *et al.* Identifying Moments of Change from Longitudinal User Text. In: *Proceedings of the 60th Annual Meeting of the Association for Computational Linguistics (Volume 1: Long Papers)*. 2022. 4647–60. doi:10.18653/V1/2022.ACL-LONG.318

44 Tsakalidis A, Chim J, Bilal IM, *et al.* Overview of the CLPsych 2022 Shared Task: Capturing Moments of Change in Longitudinal User Posts. In: *Proceedings of the Eighth Workshop on Computational Linguistics and Clinical Psychology*. Association for Computational Linguistics (ACL) 2022. 184–98. doi:10.18653/V1/2022.CLPSYCH-1.16

45 Devlin J, Chang M-W, Lee K, *et al.* BERT: Pre-training of Deep Bidirectional Transformers for Language Understanding. In: *Proceedings of the 2019 Conference of the North American Chapter of the Association for Computa-tional Linguistics: Human Language Technologies, Volume 1 (Long and Short Papers)*. 2019. 4171–86. doi:10.18653/V1/N19-1423

46 Sawhney R, Joshi H, Gandhi S, *et al.* A Time-Aware Transformer Based Model for Suicide Ideation Detection on Social Media. In: *Proceedings of the Conference Proceedings of the 2020 conference on empirical methods in natural language processing (EMNLP)*. 2020. 7685–97. doi:10.18653/V1/2020.EMNLP-MAIN.619

47 Pennebaker JW, Boyd RL, Jordan K, *et al.* *The development and psychometric properties of LIWC2015*. Austin, TX: University of Texas at Austin 2015.

48 Tsakalidis A, Liakata M, Damoulas T. Can we assess mental health through social media and smart devices? Addressing bias in methodology and evaluation. In: *European Conference on Machine Learning and Principles and Practice of Knowledge Discovery in Databases*. 2018.

For peer review only - http://bmjopen.bmj.com/site/about/guidelines.xhtml

49 Braun V, Clarke V. Using thematic analysis in psychology. *Qual Res Psychol* 2006;**3**:77–101. doi:10.1191/1478088706qp063oa

50 Williams AJ, Nielsen E, Coulson NS. 'They aren't all like that': Perceptions of clinical services, as told by self-harm online communities. *J Health Psychol* 2018;:1359105318788403. doi:10.1177/1359105318788403

51 Shanahan N, Brennan C, House A. Self-harm and social media: Thematic analysis of images posted on three social media sites. *BMJ Open* 2019;**9**. doi:10.1136/BMJOPEN-2018-027006

52 Fereday J, Muir-Cochrane E. Demonstrating Rigor Using Thematic Analysis: A Hybrid Approach of Inductive and Deductive Coding and Theme Development. *Int J Qual Methods* 2006;**5**:80–92. doi:10.1177/160940690600500107

53 O'Connor RC, Rasmussen S, Hawton K. Distinguishing adolescents who think about self-harm from those who engage in self-harm. *British Journal of Psychiatry* 2012;**200**:330–5. doi:10.1192/bjp.bp.111.097808

54 Scoliers G, Portzky G, Madge N, *et al.* Reasons for adolescent deliberate self-harm: a cry of pain and/or a cry for help? *Soc Psychiatry Psychiatr Epidemiol* 2009;**44**:601–7. doi:10.1007/s00127-008-0469-z

55 World Medical Association. World Medical Association Declaration of Helsinki: Ethical Principles for Medical Research Involving Human Subjects. American Medical Association 2013. doi:10.1001/JAMA.2013.281053

56 NHS Health Research Authority. UK Policy Framework for Health and Social Care Research - Health Research Authority. NHSHRA 2017. https://www.hra.nhs.uk/planning-and-improving-research/policies-standards-legislation/uk-policy-framework-health-social-care-research/ (accessed 3 Aug 2022).

57 General Medical Council. Confidentiality: good practice in handling patient information - ethical guidance - GMC. London: 2009. https://www.gmc-uk.org/ethical-guidance/ethical-guidance-for-doctors/confidentiality (accessed 13 Oct 2022).

58 The European Parliament and the Council Of The European Union. General Data Protection Regulation ((EU) 2016/679) (GDPR). *Official Journal of the European Union* 2016;:1–88.

59 GOV.UK. Data Protection Act, 2018. 2018.https://www.gov.uk/government/collections/data-protection-act-2018 (accessed 5 Aug 2022).

For peer review only

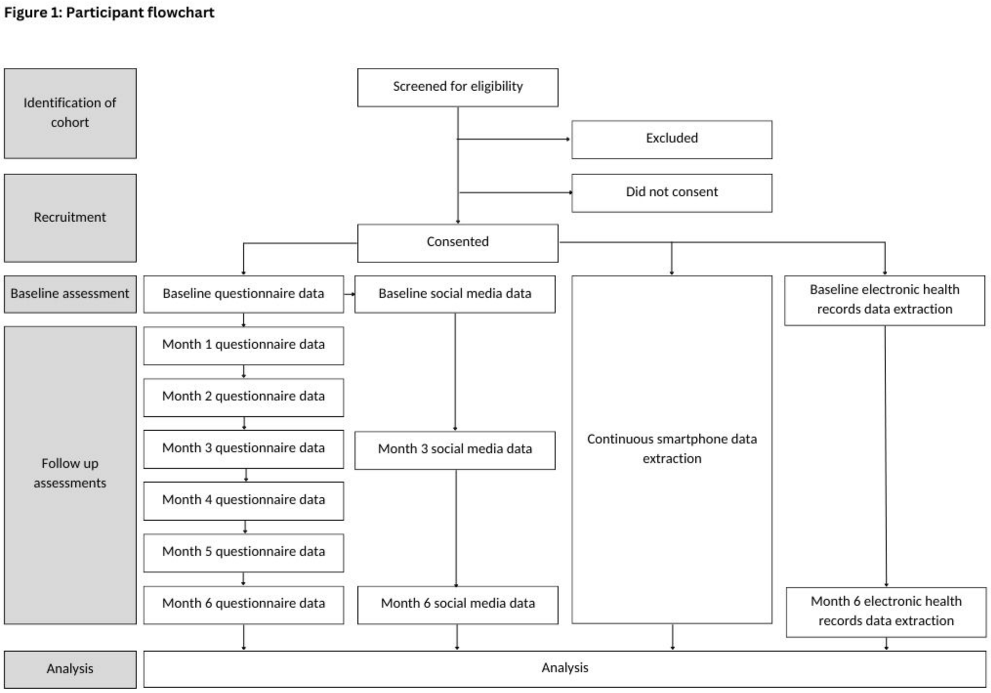

Participant flowchart

270x203mm (96 x 96 DPI)

STROBE Statement—Checklist of items that should be included in reports of ***cohort studies***

	Item No	Recommendation	Page No
**Title and abstract**	1	(*a*) Indicate the study's design with a commonly used term in the title or the abstract	1
		(*b*) Provide in the abstract an informative and balanced summary of what was done and what was found	3
**Introduction**			
Background/rationale	2	Explain the scientific background and rationale for the investigation being reported	5-6
Objectives	3	State specific objectives, including any prespecified hypotheses	6-7
**Methods**			
Study design	4	Present key elements of study design early in the paper	7
Setting	5	Describe the setting, locations, and relevant dates, including periods of recruitment, exposure, follow-up, and data collection	7
Participants	6	(*a*) Give the eligibility criteria, and the sources and methods of selection of participants. Describe methods of follow-up	7-9
		(*b*) For matched studies, give matching criteria and number of exposed and unexposed	
Variables	7	Clearly define all outcomes, exposures, predictors, potential confounders, and effect modifiers. Give diagnostic criteria, if applicable	10-14
Data sources/ measurement	8*	For each variable of interest, give sources of data and details of methods of assessment (measurement). Describe comparability of assessment methods if there is more than one group	10-13
Bias	9	Describe any efforts to address potential sources of bias	8
Study size	10	Explain how the study size was arrived at	8-9
Quantitative variables	11	Explain how quantitative variables were handled in the analyses. If applicable, describe which groupings were chosen and why	N/A
Statistical methods	12	(*a*) Describe all statistical methods, including those used to control for confounding	14-17
		(*b*) Describe any methods used to examine subgroups and interactions	14-16
		(*c*) Explain how missing data were addressed	N/A
		(*d*) If applicable, explain how loss to follow-up was addressed	N/A
		(*e*) Describe any sensitivity analyses	N/A
**Results**			
Participants	13*	(a) Report numbers of individuals at each stage of study—eg numbers potentially eligible, examined for eligibility, confirmed eligible, included in the study, completing follow-up, and analysed	N/A
		(b) Give reasons for non-participation at each stage	N/A
		(c) Consider use of a flow diagram	9
Descriptive data	14*	(a) Give characteristics of study participants (eg demographic, clinical, social) and information on exposures and potential confounders	N/A
		(b) Indicate number of participants with missing data for each variable of interest	N/A
		(c) Summarise follow-up time (eg, average and total amount)	N/A
Outcome data	15*	Report numbers of outcome events or summary measures over time	N/A

For peer review only - http://bmjopen.bmj.com/site/about/guidelines.xhtml

Main results	16	(*a*) Give unadjusted estimates and, if applicable, confounder-adjusted estimates and their precision (eg, 95% confidence interval). Make clear which confounders were adjusted for and why they were included	N/A
		(*b*) Report category boundaries when continuous variables were categorized	N/A
		(*c*) If relevant, consider translating estimates of relative risk into absolute risk for a meaningful time period	N/A
Other analyses	17	Report other analyses done—eg analyses of subgroups and interactions, and sensitivity analyses	N/A
**Discussion**			
Key results	18	Summarise key results with reference to study objectives	N/A
Limitations	19	Discuss limitations of the study, taking into account sources of potential bias or imprecision. Discuss both direction and magnitude of any potential bias	N/A
Interpretation	20	Give a cautious overall interpretation of results considering objectives, limitations, multiplicity of analyses, results from similar studies, and other relevant evidence	N/A
Generalisability	21	Discuss the generalisability (external validity) of the study results	N/A
**Other information**			
Funding	22	Give the source of funding and the role of the funders for the present study and, if applicable, for the original study on which the present article is based	19-20

*Give information separately for exposed and unexposed groups.

Note: An Explanation and Elaboration article discusses each checklist item and gives methodological background and published examples of transparent reporting. The STROBE checklist is best used in conjunction with this article (freely available on the Web sites of PLoS Medicine at http://www.plosmedicine.org/, Annals of Internal Medicine at http://www.annals.org/, and Epidemiology at http://www.epidem.com/). Information on the STROBE Initiative is available at http://www.strobe-statement.org.

For peer review only

For peer review only - http://bmjopen.bmj.com/site/about/guidelines.xhtml

BMJ Open

Observational prospective study of social media, smartphone use and self-harm in a clinical sample of young people: Study protocol

Journal:	*BMJ Open*
Manuscript ID	bmjopen-2022-069748.R1
Article Type:	Protocol
Date Submitted by the Author:	17-Jan-2023
Complete List of Authors:	Bye, Amanda; King's College London Institute of Psychiatry Psychology and Neuroscience, Psychological Medicine<br>Carter, Ben; King's College London Institute of Psychiatry Psychology and Neuroscience, Biostatistics & Health Informatics<br>Leightley, Daniel; King's College London Institute of Psychiatry Psychology and Neuroscience, Psychological Medicine; King's College London Institute of Psychiatry Psychology and Neuroscience, King's Centre for Military Health Research<br>Trevillion, Kylee; King's College London Institute of Psychiatry Psychology and Neuroscience, Health Service and Population Research<br>Liakata, Maria; Queen Mary University of London, School of Electronic Engineering & Computer Science; The Alan Turing Institute<br>Branthonne-Foster , Stella; No affiliations<br>Williamson, Grace; King's College London Institute of Psychiatry Psychology and Neuroscience, Psychological Medicine<br>Zenasni, Zohra; King's College London Institute of Psychiatry Psychology and Neuroscience, Biostatistics and Health Informatics<br>Dutta, Rina; King's College London Institute of Psychiatry Psychology and Neuroscience, Psychological Medicine; South London and Maudsley NHS Foundation Trust
**Primary Subject Heading**:	Mental health
Secondary Subject Heading:	Research methods, Qualitative research
Keywords:	Suicide & self-harm < PSYCHIATRY, Child & adolescent psychiatry < PSYCHIATRY, MENTAL HEALTH, STATISTICS & RESEARCH METHODS, QUALITATIVE RESEARCH

SCHOLARONE™
Manuscripts

TITLE

Observational prospective study of social media, smartphone use and self-harm in a clinical sample of young people: Study protocol

AUTHORS

Dr Amanda Bye (0000-0002-9808-4956)

Research Associate

Department of Psychological Medicine, Institute of Psychiatry, Psychology and Neuroscience, King's College London, London, UK

Dr Ben Carter (0000-0003-0318-8865)

Reader in Medical Statistics

Department of Biostatistics and Health Informatics, Institute of Psychiatry, Psychology and Neuroscience, King's College London, London, UK

Dr Daniel Leightley (0000-0001-9512-752X)

Research Fellow

Department of Psychological Medicine, Institute of Psychiatry, Psychology and Neuroscience, King's College London, London, UK

King's Centre for Military Health Research, Institute of Psychiatry, Psychology and Neuroscience, King's College London, London, UK

Dr Kylee Trevillion (0000-0001-5865-4726)

Senior Lecturer

Health Service and Population Research Department, Institute of Psychiatry, Psychology and Neuroscience, King's College London, London, UK

Professor Maria Liakata (0000-0001-5765-0416)

Professor in Natural Language Processing

School of Electronic Engineering & Computer Science, Queen Mary, University of London, London, UK.

The Alan Turing Institute, London, UK.

University of Warwick, UK

For peer review only

For peer review only - http://bmjopen.bmj.com/site/about/guidelines.xhtml

Ms Stella Branthonne-Foster (0000-0002-5545-9199)

Lived experience advisor

London, UK

Ms Grace Williamson (0000-0002-4618-5819)

Research Assistant

Department of Psychological Medicine, Institute of Psychiatry, Psychology and Neuroscience, King's College London, London, UK

Ms Zohra Zenasni (0000-0002-2335-1378)

Research Associate

Department of Biostatistics and Health Informatics, Institute of Psychiatry, Psychology and Neuroscience, King's College London, London, UK.

Dr Rina Dutta (0000-0002-5614-8659)

Reader in Suicidology and Psychiatry / Consultant Psychiatrist

Department of Psychological Medicine, Institute of Psychiatry, Psychology and Neuroscience, King's College London, London, UK

South London and Maudsley NHS Foundation Trust, London, UK

CORRESPONDENCE

Dr Rina Dutta

Department of Psychological Medicine, Institute of Psychiatry, Psychology and Neuroscience, King's College London, London, UK

rina.dutta@kcl.ac.uk

WORD COUNT

3,866 words

For peer review only

For peer review only - http://bmjopen.bmj.com/site/about/guidelines.xhtml

ABSTRACT

Introduction

Young people are the most frequent users of social media and smartphones and there has been increasing speculation about the potential negative impacts of their use on mental health. This has coincided with a sharp increase in the levels of self-harm in young people. To date, studies researching this potential association are predominantly cross-sectional and reliant on self-report data, which precludes the ability to objectively analyse behaviour over time. This study is one of the first attempts to explore temporal patterns of real-world usage prior to self-harm, to identify whether there are usage patterns associated with an increased risk.

Methods and analysis

To study the mechanisms by which social media and smartphone use underpin self-harm in a clinical sample of young people, the Social media, Smartphone use and Self-harm in Young People (3S-YP) study uses a prospective, observational study design. Up to 600 young people aged 13-25 years old from secondary mental health services will be recruited and followed for up to 6 months. Primary analysis will compare real-world data in the seven days leading up to a participant or clinician recorded self-harm episode, to categorise patterns of problematic usage. Secondary analyses will explore potential mediating effects of anxiety, depression, sleep disturbance, loneliness and bullying.

Ethics and dissemination

This study was approved by the National Research Ethics Service, London - Riverside, as well as by the Joint Research and Development Office of the Institute of Psychiatry, Psychology and Neuroscience and South London and Maudsley NHS Foundation Trust (SLaM), and the SLaM Clinical Research Interactive Search (CRIS) Oversight Committee. The findings from this study will be disseminated through peer-reviewed scientific journals, conferences, websites, social media and stakeholder engagement activities.

Registration details

This study is registered on ClinicalTrials.gov (ID no. NCT04601220). Pre-results.

Protocol version

Version 1.3, 09/02/2022

Keywords

For peer review only

For peer review only - http://bmjopen.bmj.com/site/about/guidelines.xhtml

Suicide & self-harm; child & adolescent psychiatry; mental health; statistics & research methods; qualitative research; social media

STRENGTHS AND LIMITATIONS

- The Social media, Smartphone use and Self-harm in Young People (3S-YP) study uses a novel prospective, observational strategy to investigate the patterns of real-world social media and smartphone usage prior to episodes of self-harm in a clinical youth sample.
- Co-production with youth experts by experience, including co-author (SBF), a leading national UK youth mental health charity – YoungMinds, and a steering group comprising of experts in the field of self-harm and suicide prevention will ensure acceptability and relevance, as well as facilitate dissemination and impact.
- Due to the online nature of the study, participants need to be able to independently engage with the digital tools and study documentation that is only available in the English language.
- The flexible approach to participation regarding which data young people choose to share, will ensure our methods are acceptable and promote autonomy among participants, though it will increase the risk of missing and inconsistent data which could compromise the validity of the results and interpretations made.
- Mixed model analyses, state-of-the-art natural language processing methods combined with thematic analysis, will be used to analyse social media metadata, textual and imagery data and smartphone metadata.

For peer review only

For peer review only - http://bmjopen.bmj.com/site/about/guidelines.xhtml

INTRODUCTION

Self-harm is characterised as any behaviour where an individual causes damage or injury to their body in response to distress. Traditionally, research has focused more on severe cases of self-harm, i.e. cutting or self-poisoning, rather than a broader spectrum of behaviour, including burning, scratching, or hitting oneself. Self-harm in young people is a significant public health concern, with findings indicating that more than 15% of young people report engaging in self-harm in the community, coupled with high rates of repetition and elevated risk of suicide.[1–3] Prevalence figures are likely to underestimate the true scale of the problem, given young people are often reluctant to disclose and seek professional help owing to fear of stigma.[4] Furthermore, epidemiological studies tend to rely on hospital presentations and only reflect those cases of self-harm that are brought to medical attention.[5] Whereas community and school-based studies collect self-reported retrospective data. Therefore, whilst capturing a wider range of self-harm behaviours, they lack information about the specific timing and nature of non-help seeking self-harm.

Reasons for self-harm are complex and multifaceted, however there has been increasing speculation about the potential negative impacts of social media and smartphone use on youth mental health.[6] Young people are the most frequent users of social media and smartphones, with 87% of 12-15 year olds using social media and 95% of 13 year olds in the UK owning a smartphone.[7] Over the last decade, social media and smartphones have altered how young people spend their time and interact socially with peers.[8] This coincides with a sharp increase in the levels of self-harm, particularly amongst adolescent females.[9] Given the widespread use of social media and smartphones, understanding the implications for youth mental health is of paramount importance.

Social media platforms are often used by people with mental health problems to share personal experiences and seek support.[10] Feedback received can be perceived as supportive by the individual.[11] Thus, social media use can alleviate loneliness and social isolation that is common amongst young people with mental health problems as it can provide opportunities for strengthening existing friendships and developing new social connections, however, it can also exacerbate loneliness if excessive time is spent online rather than time that would be otherwise spent interacting socially offline.[12,13]

Online forums have been a cause for particular concern as users may be exposed to harmful self-harm content and online discussions that normalise behaviours and discourage help-seeking, which may be associated with increased suicidal ideation found among forum users compared to other forms of

For peer review only - http://bmjopen.bmj.com/site/about/guidelines.xhtml

social media user.[10] Other studies have found significant associations between cyberbullying victimisation and self-harm in young people, and to a lesser extent, cyberbullying perpetration, likely perpetuated by the anonymous nature of social media.[14] Young people engaging in self-harm behaviours may also be more likely to engage in risky online behaviour compared to their peers.[15] More recent findings indicate that while for some young people and in certain circumstances, social media can have a positive or neutral effect, for others it can be harmful and so more research is needed to explore the effects of different types of social media use on an individual level.[16,17]

Research on the effects on mental health of excessive screen time in general is more conflicting, possibly relating to inconsistencies in measurements and definitions employed.[18–20] Sohn *et al.*,[20] found evidence of an association between problematic smartphone usage and increased anxiety and depression. Emerging evidence indicates that problematic smartphone usage may be positively linked with suicide ideation and the association may be mediated by emotional intelligence.[21,22] Furthermore, there is an established literature linking excessive smartphone use and sleep disturbance[23] and poor sleep has been linked to depression and suicidal ideation in young people,[24] as well as there being an association between sleep problems and suicidal thoughts and behaviours independent of depression.[25]

Much of the current evidence on the association between social media and smartphone use and mental health and self-harm amongst young people is limited to associations from cross-sectional studies (e.g. Mancinelli *et al*., 2021).[26] Where longitudinal studies are reported, they are based on small samples, are reliant on self-report data and have prolonged periods of time between waves of data collection (e.g. Arendt *et al*., 2019).[27] These approaches have precluded the ability to explore potential mechanisms underlying self-harm behaviour over time using real-world data, in a prospective way. This study is one of the first to attempt to explore the temporal patterns of social media and smartphone use prior to episodes of self- and clinician reported self-harm events, to identify whether there are usage patterns of social media and smartphone use that are associated with increased risk of self-harm. The findings from this study have the potential for generating wide-ranging impact as well as filling the gaps in current knowledge. The overarching aim of the 3S-YP study is to investigate the mechanisms by which social media and smartphone use underpin self-harm in a clinical sample of young people. Primary objectives are:

- To identify characteristic patterns of social media and smartphone use in the seven days preceding an episode of self-harm.

For peer review only - http://bmjopen.bmj.com/site/about/guidelines.xhtml

- To determine whether characteristic patterns of social media and smartphone use associated with an episode of self-harm, differ from young people who have not had an episode of self-harm.

Secondary objectives are to investigate whether the association is mediated by sleep disturbance, depression, anxiety, loneliness, and bullying.

METHODS AND ANALYSIS

Study design

This study is a prospective six month cohort study and has been reported according to the STROBE Checklist for Cohort Studies (see Additional file 1) (NCT04601220, protocol version 1.4, 21/07/2022).

Study setting

Young people (aged 13 to 25 years) will be recruited from South London and Maudsley NHS Foundation Trust (SLaM). SLaM is one of the largest mental healthcare providers in Europe, providing secondary mental health care to a local population of approximately 1.3 million residents as well as national and specialist services.

Participant selection

Young people will be identified via one of the following selection methods:

1) SLaM's Consent for Contact (C4C) patient research participation register of patients who have given prior consent to researchers with ethically approved studies to access their health records and contact them for the purposes of recruitment to research for which they are eligible.[28] A researcher who is independent of the study team and who works for the NIHR Biomedical Research Centre (BRC) within SLaM, will apply the eligibility criteria to the register using the Clinical Research Interactive Search (CRIS) system to screen for potentially eligible young people. The CRIS system is a case register that contains de-identified information extracted from the Trust's Electronic Health Record (EHR) system.[29] The study team will then be supplied with the Trust-identifying details of potentially eligible young people so they can check their EHRs in detail for clinical eligibility. For eligible young people, the study team will inform the care coordinator of the intention to approach prior to initiating any contact. This is so that care coordinators can advise if it is not appropriate for a young person under their care to be approached for the study.
2) If the above yields insufficient numbers, we will invite clinicians to refer young people under their care directly to the study team. We will attend team meetings to explain the study and

For peer review only - http://bmjopen.bmj.com/site/about/guidelines.xhtml

eligibility criteria to clinicians. Clinicians will screen young people under their care for eligibility and will contact eligible young people (and a parent or carer with parental responsibility for young people aged 13-15 years old) to explain the study and seek permission to share their contact details with the study team.

Eligibility criteria

Inclusion and exclusion criteria are reported in Table 1. No recruitment quotas will be imposed, with respect to any participant characteristics. To reduce the risk of participant selection bias, all eligible young people will be invited to participate until the recruitment target is met.

Table 1: Eligibility criteria

Inclusion criteria	• Identified via SLaM's C4C patient research participation register or referral to the study team by their clinician.<br>• Aged 13-25 years old at the time of study approach.<br>• Accessed mental health services at SLaM in the last 12 months.<br>• Has capacity to consent (and an adult with parental responsibility for young people aged 13-15 years old). Mental capacity will be assumed unless evidence from a clinician or during contact with the study team suggests otherwise.
Exclusion criteria	• Unable to complete the questionnaires via the study software application or online survey platform.<br>• Admitted to an inpatient psychiatric ward, sectioned under the Mental Health Act or in prison at the time of approach.<br>• Clinician advises it is not appropriate to approach.

Sample size calculation

In a recent national community survey, the reported annual age-sex incidence of self-harm for young people aged 16-18 years old was 36.4% (95%CI 22.4–52.2) for males and 43.5% (95%CI 35.5–51.7) for females.[3] Based on uptake of SLaM C4C patient research participation register, we estimate that if 600 young people consent to take part, 480 will be followed up during a 6-month period, resulting in at least 150 with an episode of self-harm. Using the most conservative estimates, if approximately 22.5% of those who do not display characteristic problematic patterns of social media usage (or smartphone usage) have an episode of self-harm and this increases to 35% in those identified with

For peer review only - http://bmjopen.bmj.com/site/about/guidelines.xhtml

problematic usage in the previous week, we expect 86% power to detect a 12.5% increase in self-harm at 5% significance, assuming 480 young people are followed up.

Recruitment method and study procedures

The study team will contact eligible young people (and/or an adult with parental responsibility for young people aged 13-15 years old) by telephone, text or email to invite the young person to take part. They will be sent a unique link to the study website which will host the participant information and online consent and assent forms as well as being offered the option of a face-to-face or virtual meeting if needed. Young people aged 16-25 years old who want to take part, will be asked to confirm their consent via an online form. This will include separate consent for the following types of data collection: questionnaires, EHRs, social media and smartphone data; choosing not to consent to the latter three will not preclude participation. For young people aged 13-15 years old, an adult with parental responsibility will first be asked to confirm their consent via an online form, following which the young person will be asked to confirm their assent. A maximum of three contact attempts will be made to non-responders. The flow of participants through the study will be documented in a flowchart (see Figure 1 - Participant flowchart).

Participants will then install the study software application on their smartphone device and complete the baseline questionnaires. The study software application has been designed to continuously extract smartphone metadata and to deliver the questionnaires for the follow-up period (up to 6 months, reduced from 12 months following an initial pilot phase). Tables 2 and 3 outlines data collection measures over the 6 month follow-up period related to primary and secondary objectives and other measurements. Participants who choose not to provide smartphone metadata (or use the study software application for any other reason) will be provided with a unique link to an online survey platform – Qualtrics (https://www.qualtrics.com) – to complete the questionnaires according to the same schedule. Participants will receive a reminder (an automated push notification and/or a pre-scheduled standardised text message and/or email) on the day a questionnaire is due, followed by a second reminder seven days later if incomplete. The final set of questionnaires may also be sent via post. Participants who consent to provide their social media data will be contacted at baseline, months 3 and 6 to ask them to upload data. Participants will be given detailed guidance on how to request their data from the platforms and upload it to the study website via a unique link. Participants will receive a £10 voucher as a thank you for enrolling in the study, followed by £5 per month of continued participation and an additional £10 voucher for uploading their social media data at month 6.

INSERT FIGURE 1 HERE

For peer review only - http://bmjopen.bmj.com/site/about/guidelines.xhtml

Table 2: Outline of data collection measures over the 6 month follow-up period related to primary objectives

Measure	Description	Data collected at:						
		Baseline	Month 1	Month 2	Month 3	Month 4	Month 5	Month 6
Socio-demographic questionnaire	Including age, gender, ethnicity, education and/or employment status, smoking, alcohol consumption and drug use.	X						
Child and Adolescent Self-harm in Europe (CASE) Study criteria [30]	Occurrence and characteristics of historical and current (i.e. in the last month) self-harm events will be collected using an adapted self-report version of the 2-item CASE Study criteria.[30]	X	X	X	X	X	X	X
Self-reported social media use	Including which social media platforms are used, types of activity (e.g. to share something, or to comment on a post), and average duration of daily usage.	X						X
Self-reported smartphone use	Including most frequently used apps, average duration of daily usage, and night-time access to smartphone.	X						X
Smartphone Addiction Scale - Short Version (SAS-SV) [31]	Smartphone addiction will also be measured using the 10-item Smartphone Addiction Scale-Short Version (SAS-SV).[SAS-SV; 31]<br>Scores range between 10 and 60, with 31 and 33 representing a cut-off for smartphone addiction in adolescent boys and girls, respectively. The SAS-SV is a widely used and validated instrument which operationalises problematic smartphone usage.[32]	X				X		X
Social media data upload	Retrospective (i.e. since the account was created) and prospective social media data will be uploaded by participants from their accounts on Facebook, Instagram, Snapchat, TikTok, Twitter and YouTube, including duration	X			X			X

For peer review only - http://bmjopen.bmj.com/site/about/guidelines.xhtml

	of usage (hours/minutes per day), timing of usage (diurnal/nocturnal posting), frequency of usage (visits to platforms per day), type of use, and textual and imagery data.							
Smartphone metadata extraction	Smartphone metadata will be extracted continuously for the duration of follow-up using the study software application, including intensity of usage by time of day, frequency and duration of incoming and outgoing telephone calls, frequency of incoming and outgoing text messages and length of time spent on software applications.<br>For participants with Android phones, permissions will be set to enable the Android App Usage API to track daily statistics listed above. For iOS, low level system logs will be used to retrieve similar information as listed above, where iOS allows.	X	X	X	X	X	X	X
Electronic health records data extraction	Clinician recorded self-harm events, using a similar approach to Polling *et al.*,[33] and other relevant clinical variables will be extracted from structured and free-text fields in EHR using the CRIS system.<br>For participants who provide social media data, data will be extracted from the date of the first interaction on social media (or the date of the thirteenth birthday, whichever is later) to the end of the follow up. For participants who do not provide social media data, data will be extracted from the date of the baseline assessment.	X						X

For peer review only

Table 3: Outline of data collection measures over the 6 month follow-up period related to secondary objectives and other measurements

Measure	Description	Data collected at:						
		Baseline	Month 1	Month 2	Month 3	Month 4	Month 5	Month 6
Generalised Anxiety Disorder (GAD-7) [34]	Symptoms of anxiety will be measured using the 7-item Generalized Anxiety Disorder Scale (GAD-7).[34]<br>Scores range between 0 and 21, with scores of 5, 10, and 15 representing the cut-off points for mild, moderate, and severe anxiety, respectively.	X				X		X
Patient Heath Questionnaire (PHQ-9) [35]	Symptoms of depression will be measured using the 9-item Patient Health Questionnaire (PHQ-9).[35]<br>Scores range between 0 and 27, with scores of 5, 10, 15, and 20 representing the cut-off points for mild, moderate, moderately severe, and severe depression, respectively.	X	X		X		X	X
PROMIS Sleep Disturbance Short Form / Pediatric Sleep Disturbance Short Form [36,37]	Symptoms of sleep disturbance will be measured using the 4-item Patient-Reported Outcomes Measurement Information System (PROMIS) Sleep Disturbance Short Form V1.0 4a (for >18 year olds) [36] or the Pediatric Sleep Disturbance Short Form V1.0 4a (for 5-17 year olds).[37]<br>Raw scores range between 4 and 16, and these are standardized using a T-score metric, with T-scores of 55, 60 and 70 representing the cut-off points for mild, moderate, and severe sleep disturbance, respectively.	X		X		X		X
Three-Item Loneliness Scale [38]	Feelings of loneliness will be measured using the 3-item Loneliness Scale.[38]<br>Scores range between 3 and 9, with higher scores indicative of greater feelings of loneliness.	X			X			X

For peer review only - http://bmjopen.bmj.com/site/about/guidelines.xhtml

Eight-item bullying checklist [39]	Experiences of bullying victimisation will be measured using the 8-item Bullying Checklist[39] derived from the Revised Olweus Bully/Victim Questionnaire (OBVQ-R).[40] Eight statements are rated using a 5-point response scale, with "2 or 3 times a month" representing a cut-off point for regular bullying victimisation.	X	X	X	X
Exposure to Covid-19 questionnaire*	Self-reported exposure to Covid-19 will be measured using 2 items from the Wave 2 survey of the Millennium Cohort Study (MCS).[41]	X			X

*Indicates that these are other measurements, not related to the primary and secondary objectives

For peer review only

For peer review only - http://bmjopen.bmj.com/site/about/guidelines.xhtml

Outcomes, exposures and other measurements

Primary outcome

Participant and clinician recorded self-harm events.

Secondary outcomes

Self-reported symptoms of anxiety, depression, sleep disturbance, loneliness and experiences of bullying victimisation.

Exposures

Social media metadata, textual and imagery data and smartphone metadata.

Self-reported social media and smartphone usage.

Other measurements

Self-reported exposure to Covid-19, sociodemographic and clinical covariates and process evaluation data.

Data analysis plan

Descriptive analysis

Individuals will be grouped into those that have had an episode of self-harm, and those that have not. Frequency distributions for different types of self-harm will be summarised, and individuals with multiple, versus a single episode of self-harm will be compared. Baseline sociodemographic and clinical descriptors will be presented for those that self-harm and those that do not. Patterns of social media and smartphone use will be explored using a mixed methods analysis and presented as a descriptive analysis.

Primary analysis

Patterns of social media and smartphone use will be identified and categorised into specific problematic exposure groups. This will be defined using a mixed methods analysis comparing social media and smartphone data in the seven days leading up to a self-harm episode. Individuals who do not experience an episode of self-harm will be randomly matched with four cases, and the same relative date and time of exposure will be randomly matched.

For peer review only - http://bmjopen.bmj.com/site/about/guidelines.xhtml

For peer review only

Social media and smartphone use will be compared between self-harm cases and non-cases, including social media platform of use, duration of use, time of use and absence of engagement (reduced level of posting but passive presence online). For example, night-time use compared to similar use during the day will be compared by self-harm status.

A binary generalized linear model with a log link will be fitted to derive the relative risk of a prospective self-harm episode in relation to a characteristic pattern of social media and smartphone use in the seven days prior to the episode. Known social and physical determinants of health associated with self-harm will be included to adjust for baseline effects. Missing data will be explored for patterns of missingness. Secondary analyses will explore potential mediating effects of anxiety, depression, sleep disturbance, loneliness and bullying.

The primary analysis population under investigation will include all participants with a post-baseline assessment of self-harm.

Natural Language Processing (NLP) and language analysis

In previous work by co-author Liakata's team[42] aggregates of both language and smartphone features were obtained at hourly intervals preceding an episode (administration of a mood form). These were used in linear and non-linear regression models for prediction of mood scores. Recent work by Liakata's team has developed NLP methods to identify changes in the mood of individuals over time through sequential analysis of two-week user timelines on social media,[43] and has shown that capturing such changes can be predictive of suicidality risk.[44] The current protocol focuses on the seven-day window preceding a self-harm episode, making the above-mentioned research directly applicable to this work. While language features used in earlier work includes word embeddings (sentiment specific and others), n-grams, topics, and lexica for sentiment, recent developments have shown the success of pre-trained language models (e.g. BERT from Devlin *et al.*, 2019)[45] in capturing information on mental health.[43,46] The textual content of individuals posts will be represented using pre-trained language models, combined with other smartphone data, to predict both moments of change and episodes of self-harm. Our earlier work on fusing heterogeneous features using multiple kernel learning,[42] will provide useful baselines. Measurements of affective change both through our models for moments of change[43] as well as more traditional measures of valence and affect[47] will feed into the predictive models as well as the descriptive analysis. Models will be evaluated under robust cross validation settings to simulate as much as possible a real-world scenario and avoid overfitting.[48]

For peer review only - http://bmjopen.bmj.com/site/about/guidelines.xhtml

Qualitative analysis

Thematic analysis,[49] which has been successfully applied in the analysis of social media data of patients and self-harm online communities,[50,51] will be employed to explore characteristic patterns of social media use in the seven days leading up to a self-harm episode from a qualitative perspective, complimentary to the main analyses. Thematic analysis comprises six stages: (1) familiarisation with the data; (2) generation of initial codes; (3) development of themes; (4) review of themes; and (5) defining themes; and (6) writing up the findings.[49]

Data from a purposive sample of cases will be selected for this analysis, based on age, sex, ethnicity, frequency of social media usage around the time of a self-harm episode and type of self-harm behaviour. Consistent with related work in this field (e.g. Shanahan *et al*., 2019; Williams *et al*., 2018)[50,51] around 500-600 social media postings will be analysed. Following removal of any personally identifiable/geographical material and duplicate material (for example, re-tweets on Twitter), the textual and imagery data of social media posts in the seven days leading up to and including an episode of self-harm will be analysed using inductive and deductive methods to validate and refine the emerging themes.[52] Inductive methods comprise repeated reading of the data to identify salient themes and deductive methods involve testing out pre-defined themes based on the research question and existing theories, for example, cry of pain theory/integrated motivational-volitional model.[53,54] Analysis of visual data will include descriptive summaries of the visual content as well as analysis of thematic content. Coding frames for the analysis of textual and visual data will be developed by the study team, informed by relevant published literature (e.g. Shanahan *et al*., 2019)[51] the research question and pre-defined themes. The coding frames will be refined deductively throughout the analytical process.

For peer review only

Process evaluation

Data from participant contact will be collected by the study team to evaluate the study processes for acceptability, feasibility and value. Reasons for decline and withdrawal will be coded and categorised where appropriate and summarised using descriptive statistics. A purposive sample of up to 20 participants (who provided consent to be contacted for further research when they enrolled in the 3S-YP study) will be invited to take part in a brief telephone or in person interview. Participants will complete a separate consent form and interviews will take place following study completion or drop out from the study (including participants who withdraw or stop providing data to the study). Interviews will be audio recorded and transcribed verbatim. Participants will receive a £20 voucher as

For peer review only - http://bmjopen.bmj.com/site/about/guidelines.xhtml

a thank you for taking part. Interview data will be analysed using Braun and Clark's[49] thematic analysis approach, as outlined above.

ETHICS AND DISSEMINATION

Ethics and safety considerations

This study is being conducted in compliance with the principles of the Declaration of Helsinki[55] and Good Clinical Practice as outlined in the UK Policy Framework for Health and Social Care Research.[56]

This study was approved by the National Research Ethics Service, London – Riverside (ethics ref 20/LO/1187; IRAS ref 269104), as well as by the Joint Research and Development Office of the Institute of Psychiatry, Psychology and Neuroscience and SLaM and the SLaM CRIS Oversight Committee (ref 20-074 and 21-039). The SLaM C4C patient research participation register was approved by the National Research Ethics Service, London – South East (ref 10/H0807/88), as well as by the SLaM Caldicott Guardian and the SLaM Trust Executive. The CRIS system was approved as a dataset for secondary data analysis by the National Research Ethics Service, South Central – Oxford C (08/H06060/71). This study is registered on ClinicalTrials.gov (ref NCT04601220).

Given the observational nature of the study, participation in this study is not anticipated to contribute to any specific adverse events. Drawing on our previous research and through careful planning with youth experts by experience, we have developed detailed procedures for conducting the study in a manner that is sensitive to the needs of young people, while maintaining high scientific standards and addressing any arising risks. Young people will provide their informed consent (or assent for young people aged 13-15 years old, along with consent from an adult with parental responsibility) prior to recruitment to the study. Participants can choose what data they want to share. Participants will be informed of the limits of confidentiality and their right to withdraw. Participants will be able to skip questions, take a break, or terminate a questionnaire at any time. Participants will be provided with details of resources for accessing support and the study team's contact information in the event of problems with any aspect of participation. The study team will have received training in the principles of Good Clinical Practice, informed consent, identifying and managing distress and risk (along with receiving regular supervision from the study chief investigator, RD), and the technical aspects of participation.

For peer review only - http://bmjopen.bmj.com/site/about/guidelines.xhtml

Data will be handled in accordance with the General Medical Council guidance on confidentiality,[57] the principles of Good Clinical Practice, European General Data Protection Regulations[58] and UK Data Protection Act.[59]

Dissemination plan

Data collected in the study will not be made publicly available as it is not possible to guarantee that an individual could not be identified as a participant from their publicly available social media data. The findings will be disseminated through peer-reviewed scientific journals, conferences, websites, social media and stakeholder engagement activities.

PROJECT OVERSIGHT

Project oversight is provided by our steering group, comprised of researchers and clinicians from across the fields of self-harm and suicide prevention, CAMHS, data science, public health, sleep medicine, cyberbullying and addictions, as well as young people with lived experience, representatives from the leading national UK youth mental health charity – YoungMinds, and communications and funding advisors. The group will meet at regular intervals throughout the study. The role of the group is to monitor study conduct and progress and contribute to decision making and solution generation. The group will not have access to participants' personal data.

PATIENT AND PUBLIC INVOLVEMENT

Co-author (SBF), a senior service user consultant, and representatives from YoungMinds are facilitating an embedded participatory research approach to promote youth engagement and representation. Together we have developed safe working practices to ensure young people with lived experience are supported and able to contribute to the various engagement activities. Research priorities and consideration of key ethical issues were identified at the pre-funding stage, through a series of consultations and workshops with youth service users from SLaM. When the study was being set-up, with support from YoungMinds, we partnered with two youth advisors who joined the steering group and contribute to co-production for the study duration. Study procedures and participant-facing digital tools and documentation were co-designed through consultations with our youth advisors and a workshop facilitated by YoungMinds with other young people to ensure the procedures and materials are acceptable and inclusive from a range of perspectives. Towards the end of data collection, we will conduct further engagement activities with YoungMinds to inform the analysis and dissemination plans.

For peer review only - http://bmjopen.bmj.com/site/about/guidelines.xhtml

For peer review only

STUDY STATUS

The study commenced recruitment in June 2021 and aims to conclude data collection in 2023.

AVAILABILITY OF DATA AND MATERIALS

Not applicable – this manuscript does not contain any data.

ACKNOWLEDGEMENTS

We would like to thank all the members of the steering group, including Louise Arsenault, Janis Baird, Johnny Downs, Tamsin Ford, Nuala Flewett, Paul Gringras, Matthew Hotopf, Hazel Inskip, Nicola Kalk, Navneet Kapur, Fiona Lacey, Dennis Ougrin, Louise Pratt, Angus Roberts, Edmund Sonuga-Barke, Robert Stewart, Jack Stonebridge, Alastair Sutcliffe, Sumithra Velupillai and Tony Wood, and the young people who have been involved in co-production activities for their invaluable contributions to the development of the 3S-YP study. We would like to thank YoungMinds for partnering with us. We would also like to thank local NIHR CRN South London staff for support in recruiting participants.

AUTHOR CONTRIBUTIONS

RD, BC, KT, SBF and ML contributed to the conception of the study and obtaining the funding. RD is the study CI. RD, BC, KT, SBF, ML, DL and AB contributed to the design of the study. BC performed the power analysis. RD, BC, KT, SBF, ML, DL, AB and ZZ contributed to the analysis plans. DL, AB, SBF, RD and GW contributed to the development or testing of the systems and software application used for data collection and management. DL, AB and RD contributed to the development of the data protection and storage systems. AB prepared the protocol and other study documentation for ethical approval and the first draft of the manuscript for publication, and is responsible for the study management, with oversight by RD. All authors contributed to revising the manuscript and approved the final version to be published. All authors have agreed to be accountable for all aspects of the work in ensuring that questions related to the accuracy or integrity of any part of the work are appropriately investigated and resolved.

FUNDING STATEMENT

This paper represents independent research supported by the Medical Research Council and Medical Research Foundation (grant number MR/S020365/1). This research was also part supported by the National Institute for Health Research (NIHR) Biomedical Research Centre at South London and Maudsley NHS Foundation Trust and King's College London. RD was also funded by a Clinician Scientist Fellowship from the Health Foundation in partnership with the Academy of Medical Sciences and her

For peer review only - http://bmjopen.bmj.com/site/about/guidelines.xhtml

work is supported by the National Institute for Health Research (NIHR) Biomedical Research Centre at South London and Maudsley NHS Foundation Trust and King's College London. BC is also supported by the Nuffield Trust. ML is also supported by the Engineering and Physical Sciences Research Council (grant number EP/V030302/1) and The Alan Turing Institute (grant number EP/N510129/1). DL is also supported by the Forces in Mind Trust (Project number: FIMT/0323KCL), a funding scheme run by the Forces in Mind Trust using an endowment awarded by the National Lottery Community Fund. The views expressed are those of the author(s) and not necessarily those of the MRC, the MRF, the NHS, the NIHR or the Department of Health and Social Care.

COMPETING INTERESTS STATEMENT

None of the authors declares a conflict of interest.

PATIENT CONSENT REQUIRED FOR PUBLICATION

Not required.

REFERENCES

1 Hawton K, Bergen H, Kapur N, *et al.* Repetition of self-harm and suicide following self-harm in children and adolescents: findings from the Multicentre Study of Self-harm in England. *Journal of Child Psychology and Psychiatry* 2012;**53**:1212–9. doi:10.1111/J.1469-7610.2012.02559.X

2 Bennardi M, McMahon E, Corcoran P, *et al.* Risk of repeated self-harm and associated factors in children, adolescents and young adults. *BMC Psychiatry* 2016;**16**:1–12. doi:10.1186/s12888-016-1120-2

3 Morey Y, Mellon D, Dailami N, *et al.* Adolescent self-harm in the community: an update on prevalence using a self-report survey of adolescents aged 13-18 in England. *J Public Health (Oxf)* 2017;**39**:58–64. doi:10.1093/pubmed/fdw010

4 Rowe SL, French RS, Henderson C, *et al.* Help-seeking behaviour and adolescent self-harm: A systematic review. *Australian and New Zealand Journal of Psychiatry* 2014;**48**:1083–95. doi:10.1177/0004867414555718

5 Hawton K, Saunders KEA, O'Connor RC. Self-harm and suicide in adolescents. The Lancet. 2012;**379**:2373–82. doi:10.1016/S0140-6736(12)60322-5

6 Abi-Jaoude E, Naylor KT, Pignatiello A. Smartphones, social media use and youth mental health. *CMAJ* 2020;**192**:E136–41. doi:10.1503/CMAJ.190434

7 Ofcom. Children and parents: media use and attitudes report Content consumption and online activities. 2021. https://www.ofcom.org.uk/__data/assets/pdf_file/0025/217825/children-and-parents-media-use-and-attitudes-report-2020-21.pdf (accessed 14 Oct 2022).

For peer review only - http://bmjopen.bmj.com/site/about/guidelines.xhtml

8 Twenge JM, Spitzberg BH, Campbell WK. Less in-person social interaction with peers among U.S. adolescents in the 21st century and links to loneliness. *J Soc Pers Relat* 2019;**36**:1892–913. doi:10.1177/0265407519836170

9 Morgan C, Webb RT, Carr MJ, *et al.* Incidence, clinical management, and mortality risk following self harm among children and adolescents: cohort study in primary care. *BMJ* 2017;**359**:j4351. doi:10.1136/bmj.j4351

10 Daine K, Hawton K, Singaravelu V, *et al.* The Power of the Web: A Systematic Review of Studies of the Influence of the Internet on Self-Harm and Suicide in Young People. *PLoS One* 2013;**8**:e77555. doi:10.1371/journal.pone.0077555

11 de Choudhury M, De S. Mental Health Discourse on reddit: Self-Disclosure, Social Support, and Anonymity. *Proceedings of the Eighth International AAAI Conference on Weblogs and Social Media* 2014;**8**:71–80.

12 Nowland R, Necka EA, Cacioppo JT. Loneliness and Social Internet Use: Pathways to Reconnection in a Digital World? *https://doi.org/101177/1745691617713052* 2017;**13**:70–87. doi:10.1177/1745691617713052

13 Matthews T, Danese A, Caspi A, *et al.* Lonely young adults in modern Britain: findings from an epidemiological cohort study. *Psychol Med* 2019;**49**:268–77. doi:10.1017/S0033291718000788

14 John A, Glendenning AC, Marchant A, *et al.* Self-Harm, Suicidal Behaviours, and Cyberbullying in Children and Young People: Systematic Review. *J Med Internet Res* 2018;**20**. doi:10.2196/JMIR.9044

15 Mitchell KJ, Ybarra ML. Online behavior of youth who engage in self-harm provides clues for preventive intervention. *Prev Med (Baltim)* 2007;**45**:392–6. doi:10.1016/J.YPMED.2007.05.008

16 Beyens I, Loes Pouwels J, van Driel II, *et al.* Social Media Use and Adolescents' Well-Being: Developing a Typology of Person-Specific Effect Patterns. *Science Reports* 2020;**10**. doi:10.1177/00936502211038196

17 Sedgwick R, Epstein S, Dutta R, *et al.* Social media, internet use and suicide attempts in adolescents. *Curr Opin Psychiatry* 2019;**32**:534–41. doi:10.1097/YCO.0000000000000547

18 Orben A, Przybylski AK. The association between adolescent well-being and digital technology use. *Nat Hum Behav* 2019;**3**:173–82. doi:10.1038/S41562-018-0506-1

19 Orben A, Przybylski AK. Corrigendum: Screens, Teens, and Psychological Well-Being: Evidence From Three Time-Use-Diary Studies. *Psychol Sci* 2019;**30**:682–696. doi:10.1177/0956797619862548

20 Sohn S, Rees P, Wildridge B, *et al.* Prevalence of problematic smartphone usage and associated mental health outcomes amongst children and young people: a systematic review, meta-analysis and GRADE of the evidence. *BMC Psychiatry* 2019;**19**:356. doi:10.1186/s12888-019-2350-x

21 Arrivillaga C, Rey L, Extremera N. Adolescents' problematic internet and smartphone use is related to suicide ideation: Does emotional intelligence make a difference? *Comput Human Behav* 2020;**110**:106375. doi:10.1016/j.chb.2020.106375

For peer review only

For peer review only - http://bmjopen.bmj.com/site/about/guidelines.xhtml

22 Lung F-W, Shu B-C, Chiang T-L, *et al.* Relationships between internet use, deliberate self-harm, and happiness in adolescents: A Taiwan birth cohort pilot study. *PLoS One* 2020;**15**:e0235834. doi:10.1371/journal.pone.0235834

23 Carter B, Rees P, Hale L, *et al.* Association Between Portable Screen-Based Media Device Access or Use and Sleep Outcomes A Systematic Review and Meta-analysis. *JAMA Pediatr* 2016;**170**:1202–8. doi:10.1001/jamapediatrics.2016.2341

24 Gangwisch JE, Babiss LA, Malaspina D, *et al.* Earlier parental set bedtimes as a protective factor against depression and suicidal ideation. *Sleep* 2010;**33**:97–106. doi:10.1093/SLEEP/33.1.97

25 Pigeon WR, Pinquart M, Conner K. Meta-analysis of sleep disturbance and suicidal thoughts and behaviors. *J Clin Psychiatry* 2012;**73**. doi:10.4088/JCP.11R07586

26 Mancinelli E, Sharka O, Lai T, *et al.* Self-injury and Smartphone Addiction: Age and gender differences in a community sample of adolescents presenting self-injurious behavior. *Health Psychol Open* 2021;**8**. doi:10.1177/20551029211038811

27 Arendt F, Scherr S, Romer D. Effects of exposure to self-harm on social media: Evidence from a two-wave panel study among young adults. *New Media Soc* 2019;**21**:2422–42. doi:10.1177/1461444819850106

28 Callard F, Broadbent M, Denis M, *et al.* Developing a new model for patient recruitment in mental health services: a cohort study using Electronic Health Records. *BMJ Open* 2014;**4**:5654. doi:10.1136/bmjopen-2014-005654

29 Perera G, Broadbent M, Callard F, *et al.* Cohort profile of the South London and Maudsley NHS Foundation Trust Biomedical Research Centre (SLaM BRC) Case Register: current status and recent enhancement of an Electronic Mental Health Record-derived data resource. *BMJ Open* 2016;**6**:8721. doi:10.1136/bmjopen-2015

30 Madge N, Hewitt A, Hawton K, *et al.* Deliberate self-harm within an international community sample of young people: comparative findings from the Child & Adolescent Self-harm in Europe (CASE) Study. *Journal of Child Psychology and Psychiatry* 2008;**49**:667–77. doi:10.1111/j.1469-7610.2008.01879.x

31 Kwon M, Kim D-J, Cho H, *et al.* The Smartphone Addiction Scale: Development and Validation of a Short Version for Adolescents. *PLoS One* 2013;**8**:e83558. doi:10.1371/journal.pone.0083558

32 Olson JA, Sandra DA, Colucci ÉS, *et al.* Smartphone addiction is increasing across the world: A meta-analysis of 24 countries. *Comput Human Behav* 2022;**129**:107138. doi:10.1016/J.CHB.2021.107138

33 Polling C, Tulloch A, Banerjee S, *et al.* Using routine clinical and administrative data to produce a dataset of attendances at Emergency Departments following self-harm. *BMC Emerg Med* 2015;**15**:1–8. doi:10.1186/S12873-015-0041-6/TABLES/4

34 Spitzer RL, Kroenke K, Williams JBW, *et al.* A Brief Measure for Assessing Generalized Anxiety Disorder. *Arch Intern Med* 2006;**166**:1092. doi:10.1001/archinte.166.10.1092

35 Kroenke K, Spitzer RL, Williams JBW. The PHQ-9: Validity of a brief depression severity measure. *J Gen Intern Med* 2001;**16**:606–13. doi:10.1046/j.1525-1497.2001.016009606.x

For peer review only

36 Yu L, Buysse DJ, Germain A, *et al.* Development of Short Forms From the PROMIS™ Sleep Disturbance and Sleep-Related Impairment Item Banks. *Behavioral Sleep Medicine* 2011;**10**:6–24. doi:10.1080/15402002.2012.636266

37 Forrest CB, Meltzer LJ, Marcus CL, *et al.* Development and validation of the PROMIS Pediatric Sleep Disturbance and Sleep-Related Impairment item banks. *Sleep* 2018;**41**:1–13. doi:10.1093/sleep/zsy054

38 Hughes ME, Waite LJ, Hawkley LC, *et al.* A Short Scale for Measuring Loneliness in Large Surveys: Results From Two Population-Based Studies. *Res Aging* 1999;**26**:655–72.

39 Przybylski AK, Bowes L. Cyberbullying and adolescent well-being in England: a population-based cross-sectional study. *Lancet Child Adolesc Health* 2017;**1**:19–26. doi:10.1016/S2352-4642(17)30011-1

40 Olweus D. Revised Olweus Bully/Victim Questionnaire. 1996;:0–12. doi:10.1037/t09634-000

41 University College London UI of EC for LS. COVID-19 Survey in Five National Longitudinal Cohort Studies: Millennium Cohort Study, Next Steps, 1970 British Cohort Study and 1958 National Child Development Study, 2020-2021. 4th Edition. UK Data Service. SN: 8658. 2022.

42 Tsakalidis A, Liakata M, Damoulas T, *et al.* Combining Heterogeneous User Generated Data to Sense Well-being. In: *Proceedings of COLING 2016, the 26th International Conference on Computational Linguistics : Technical Papers*. 2016. 3007–18.http://wing.comp.nus.edu.sg:8080/SMSCorpus/contribution.jsp (accessed 5 Jun 2020).

43 Tsakalidis A, Nanni F, Hills A, *et al.* Identifying Moments of Change from Longitudinal User Text. In: *Proceedings of the 60th Annual Meeting of the Association for Computational Linguistics (Volume 1: Long Papers)*. 2022. 4647–60. doi:10.18653/V1/2022.ACL-LONG.318

44 Tsakalidis A, Chim J, Bilal IM, *et al.* Overview of the CLPsych 2022 Shared Task: Capturing Moments of Change in Longitudinal User Posts. In: *Proceedings of the Eighth Workshop on Computational Linguistics and Clinical Psychology*. Association for Computational Linguistics (ACL) 2022. 184–98. doi:10.18653/V1/2022.CLPSYCH-1.16

45 Devlin J, Chang M-W, Lee K, *et al.* BERT: Pre-training of Deep Bidirectional Transformers for Language Understanding. In: *Proceedings of the 2019 Conference of the North American Chapter of the Association for Computa-tional Linguistics: Human Language Technologies, Volume 1 (Long and Short Papers)*. 2019. 4171–86. doi:10.18653/V1/N19-1423

46 Sawhney R, Joshi H, Gandhi S, *et al.* A Time-Aware Transformer Based Model for Suicide Ideation Detection on Social Media. In: *Proceedings of the Conference Proceedings of the 2020 conference on empirical methods in natural language processing (EMNLP)*. 2020. 7685–97. doi:10.18653/V1/2020.EMNLP-MAIN.619

47 Pennebaker JW, Boyd RL, Jordan K, *et al.* *The development and psychometric properties of LIWC2015*. Austin, TX: University of Texas at Austin 2015.

48 Tsakalidis A, Liakata M, Damoulas T. Can we assess mental health through social media and smart devices? Addressing bias in methodology and evaluation. In: *European Conference on Machine Learning and Principles and Practice of Knowledge Discovery in Databases*. 2018.

49 Braun V, Clarke V. Using thematic analysis in psychology. *Qual Res Psychol* 2006;**3**:77–101. doi:10.1191/1478088706qp063oa

For peer review only - http://bmjopen.bmj.com/site/about/guidelines.xhtml

50 Williams AJ, Nielsen E, Coulson NS. 'They aren't all like that': Perceptions of clinical services, as told by self-harm online communities. *J Health Psychol* 2018;:1359105318788403. doi:10.1177/1359105318788403

51 Shanahan N, Brennan C, House A. Self-harm and social media: Thematic analysis of images posted on three social media sites. *BMJ Open* 2019;**9**. doi:10.1136/BMJOPEN-2018-027006

52 Fereday J, Muir-Cochrane E. Demonstrating Rigor Using Thematic Analysis: A Hybrid Approach of Inductive and Deductive Coding and Theme Development. *Int J Qual Methods* 2006;**5**:80–92. doi:10.1177/160940690600500107

53 O'Connor RC, Rasmussen S, Hawton K. Distinguishing adolescents who think about self-harm from those who engage in self-harm. *British Journal of Psychiatry* 2012;**200**:330–5. doi:10.1192/bjp.bp.111.097808

54 Scoliers G, Portzky G, Madge N, *et al.* Reasons for adolescent deliberate self-harm: a cry of pain and/or a cry for help? *Soc Psychiatry Psychiatr Epidemiol* 2009;**44**:601–7. doi:10.1007/s00127-008-0469-z

55 World Medical Association. World Medical Association Declaration of Helsinki: Ethical Principles for Medical Research Involving Human Subjects. American Medical Association 2013. doi:10.1001/JAMA.2013.281053

56 NHS Health Research Authority. UK Policy Framework for Health and Social Care Research - Health Research Authority. NHSHRA 2017. https://www.hra.nhs.uk/planning-and-improving-research/policies-standards-legislation/uk-policy-framework-health-social-care-research/ (accessed 3 Aug 2022).

57 General Medical Council. Confidentiality: good practice in handling patient information - ethical guidance - GMC. London: 2009. https://www.gmc-uk.org/ethical-guidance/ethical-guidance-for-doctors/confidentiality (accessed 13 Oct 2022).

58 The European Parliament and the Council Of The European Union. General Data Protection Regulation ((EU) 2016/679) (GDPR). *Official Journal of the European Union* 2016;:1–88.

59 GOV.UK. Data Protection Act, 2018. 2018.https://www.gov.uk/government/collections/data-protection-act-2018 (accessed 5 Aug 2022).

For peer review only

For peer review only - http://bmjopen.bmj.com/site/about/guidelines.xhtml

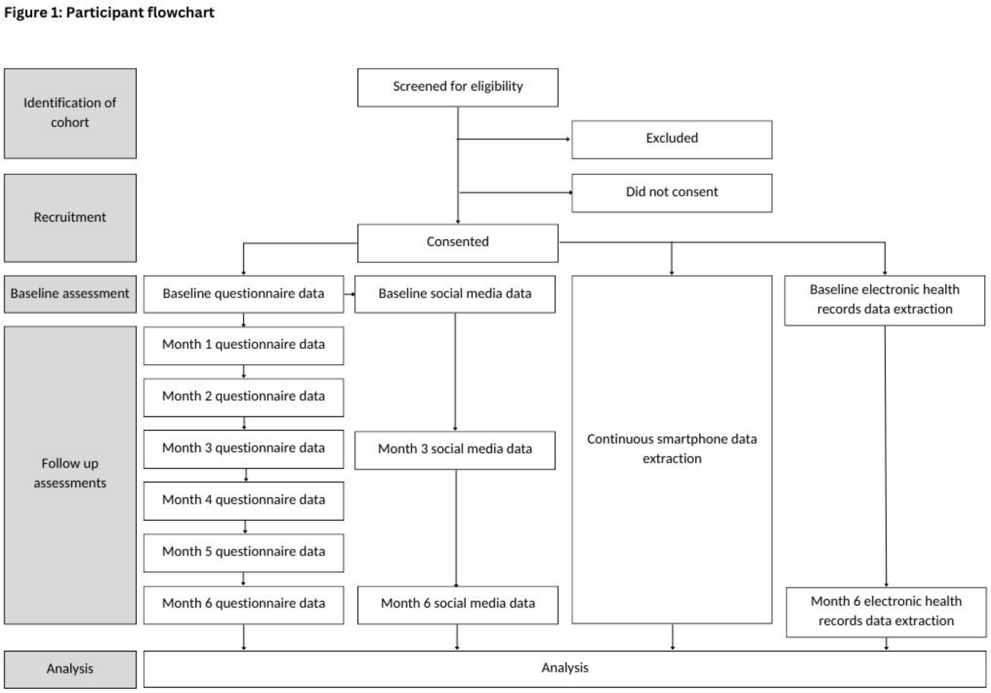

Participant flowchart

270x203mm (96 x 96 DPI)

STROBE Statement—Checklist of items that should be included in reports of ***cohort studies***

	Item No	Recommendation	Page No
**Title and abstract**	1	(*a*) Indicate the study's design with a commonly used term in the title or the abstract	1
		(*b*) Provide in the abstract an informative and balanced summary of what was done and what was found	3
**Introduction**			
Background/rationale	2	Explain the scientific background and rationale for the investigation being reported	5-6
Objectives	3	State specific objectives, including any prespecified hypotheses	6-7
**Methods**			
Study design	4	Present key elements of study design early in the paper	7
Setting	5	Describe the setting, locations, and relevant dates, including periods of recruitment, exposure, follow-up, and data collection	7
Participants	6	(*a*) Give the eligibility criteria, and the sources and methods of selection of participants. Describe methods of follow-up	7-9
		(*b*) For matched studies, give matching criteria and number of exposed and unexposed	
Variables	7	Clearly define all outcomes, exposures, predictors, potential confounders, and effect modifiers. Give diagnostic criteria, if applicable	10-14
Data sources/ measurement	8*	For each variable of interest, give sources of data and details of methods of assessment (measurement). Describe comparability of assessment methods if there is more than one group	10-13
Bias	9	Describe any efforts to address potential sources of bias	8
Study size	10	Explain how the study size was arrived at	8-9
Quantitative variables	11	Explain how quantitative variables were handled in the analyses. If applicable, describe which groupings were chosen and why	N/A
Statistical methods	12	(*a*) Describe all statistical methods, including those used to control for confounding	14-17
		(*b*) Describe any methods used to examine subgroups and interactions	14-16
		(*c*) Explain how missing data were addressed	N/A
		(*d*) If applicable, explain how loss to follow-up was addressed	N/A
		(*e*) Describe any sensitivity analyses	N/A
**Results**			
Participants	13*	(a) Report numbers of individuals at each stage of study—eg numbers potentially eligible, examined for eligibility, confirmed eligible, included in the study, completing follow-up, and analysed	N/A
		(b) Give reasons for non-participation at each stage	N/A
		(c) Consider use of a flow diagram	9
Descriptive data	14*	(a) Give characteristics of study participants (eg demographic, clinical, social) and information on exposures and potential confounders	N/A
		(b) Indicate number of participants with missing data for each variable of interest	N/A
		(c) Summarise follow-up time (eg, average and total amount)	N/A
Outcome data	15*	Report numbers of outcome events or summary measures over time	N/A

For peer review only - http://bmjopen.bmj.com/site/about/guidelines.xhtml

Main results	16	(*a*) Give unadjusted estimates and, if applicable, confounder-adjusted estimates and their precision (eg, 95% confidence interval). Make clear which confounders were adjusted for and why they were included	N/A
		(*b*) Report category boundaries when continuous variables were categorized	N/A
		(*c*) If relevant, consider translating estimates of relative risk into absolute risk for a meaningful time period	N/A
Other analyses	17	Report other analyses done—eg analyses of subgroups and interactions, and sensitivity analyses	N/A
**Discussion**			
Key results	18	Summarise key results with reference to study objectives	N/A
Limitations	19	Discuss limitations of the study, taking into account sources of potential bias or imprecision. Discuss both direction and magnitude of any potential bias	N/A
Interpretation	20	Give a cautious overall interpretation of results considering objectives, limitations, multiplicity of analyses, results from similar studies, and other relevant evidence	N/A
Generalisability	21	Discuss the generalisability (external validity) of the study results	N/A
**Other information**			
Funding	22	Give the source of funding and the role of the funders for the present study and, if applicable, for the original study on which the present article is based	19-20

*Give information separately for exposed and unexposed groups.

Note: An Explanation and Elaboration article discusses each checklist item and gives methodological background and published examples of transparent reporting. The STROBE checklist is best used in conjunction with this article (freely available on the Web sites of PLoS Medicine at http://www.plosmedicine.org/, Annals of Internal Medicine at http://www.annals.org/, and Epidemiology at http://www.epidem.com/). Information on the STROBE Initiative is available at http://www.strobe-statement.org.

For peer review only
