# Supplementary material for: Observational prospective study of social media, smartphone use and self-harm in a clinical sample of young people: study protocol
Source: BMJ Open. 2023 Feb 1;13(2):e069748. doi: 10.1136/bmjopen-2022-069748 (PMC9896249; doi:10.1136/bmjopen-2022-069748)
Supplement: Reviewer comments [file bmjopen-2022-069748.reviewer_comments.pdf]

PEER REVIEW HISTORY

BMJ Open publishes all reviews undertaken for accepted manuscripts. Reviewers are asked to complete a checklist review form (**http://bmjopen.bmj.com/site/about/resources/checklist.pdf**) and are provided with free text boxes to elaborate on their assessment. These free text comments are reproduced below.

ARTICLE DETAILS

**TITLE (PROVISIONAL)**	Observational prospective study of social media, smartphone use and self-harm in a clinical sample of young people: Study protocol
**AUTHORS**	Bye, Amanda; Carter, Ben; Leightley, Daniel; Trevillion, Kylee; Liakata, Maria; Branthonne-Foster, Stella; Williamson, Grace; Zenasni, Zohra; Dutta, Rina

This article was not externally reviewed at BMJ Open. Protocols that have been independently assessed prior to submission to BMJ Open are usually fast-tracked to publication on the grounds that further substantial changes will not be possible. This independent assessment will usually be external, independent review for both a substantial grant award from a non-commercial or government funder and ethics approval.
